# A variable sampling interval run sum chart for monitoring multivariate coefficient of variation

**DOI:** 10.1371/journal.pone.0330936

**Published:** 2025-09-23

**Authors:** Dongmei Cui, Michael B. C. Khoo, Sajal Saha, Zhi Lin Chong, Shanyu Chua

**Affiliations:** 1 School of Business, Hunan International Economics University, Changsha, Hunan, China; 2 School of Mathematical Sciences, Universiti Sains Malaysia, Minden, Penang, Malaysia; 3 Department of Mathematics, International University of Business Agriculture and Technology, Dhaka, Bangladesh; 4 Department of Electronic Engineering, Faculty of Engineering and Green Technology, Universiti Tunku Abdul Rahman, Kampar, Malaysia; 5 Estek Automation Sdn Bhd, Bayan Lepas, Penang, Malaysia; University of Ilorin, NIGERIA

## Abstract

The coefficient of variation (CV) is employed to develop control charts to measure the relative dispersion of the data. The multivariate coefficient of variation (MCV) chart is used to monitor the CV in Phase-II in a multivariate framework. In this paper, the upward and downward variable sampling interval run sum multivariate coefficient of variation (VSI RS MCV) charts are developed to detect MCV shifts. The developed VSI RS MCV charts are evaluated and compared with their existing MCV and RS MCV counterparts using the average time to signal (ATS), standard deviation of the time to signal (SDTS) and expected average time to signal (EATS) criteria. Optimization programs incorporating the Markov chain methodology are developed in MATLAB to compute the optimal parameters and scores of the developed VSI RS MCV charts that minimize the charts’ out-of-control ATS or EATS value. The findings show that the developed VSI RS MCV charts outperform both the existing RS MCV and MCV charts, for all shift sizes, in terms of the out-of-control ATS, SDTS and EATS criteria. An example is provided to elucidate the implementation of the proposed VSI RS MCV charts.

## 1. Introduction

A control chart is used for monitoring and controlling the quality of a production process. It plays a critical role in maintaining product quality by detecting process shifts. In usual circumstances, an in-control process will have a constant mean and variance and control charts are usually used to monitor either the process mean or variation. In cases when both the process mean and variation are not constants even when the process is in-control, an important metric often used in statistical process control (SPC) is the coefficient of variation (CV), which is the ratio of the standard deviation to the mean, i.e., σ/μ.  The first CV chart for monitoring the sample CV was proposed by [1]. Ayesha et al. [2] suggested an adaptive EWMA CV chart employed in the sintering process, where exceptional monitoring performance was demonstrated. In the multivariate context, where several quality characteristics are monitored simultaneously, the multivariate coefficient of variation (MCV) type charts serve as important tools for assessing the variability in the process MCV. Yeong et al. [3] developed the first MCV chart. Subsequently, Abbasi and Adegoke [4] discussed the application of MCV charts in Phase-I process monitoring. Khaw et al. [5] extended this work by introducing adaptive control charts for monitoring the MCV, where the charts’ parameters are dynamically adjusted based on the current process quality. Chew et al. [6] introduced a variable parameters MCV chart, which further enhances the flexibility of designing the MCV chart. In order to further increase the sensitivity of MCV charts in detecting small shifts, Yeong et al. [7] suggested the side-sensitive synthetic chart. Saha et al. [8] proposed a specialized multivariate run sum control chart to address the problems of autocorrelation in the basic multivariate run sum chart. To further augment the flexibility of MCV charts for monitoring small batch manufacturing processes, Hu et al. [9] introduced two one-sided cumulative sum MCV schemes for detecting deterministic and random shifts.

The run sum (RS) charts work by accumulating scores and trigger an out-of-control signal when the accumulated scores exceed a specified threshold. The RS charts are particularly useful in detecting small and moderate shifts. The pioneering work on the RS chart was conducted by [10], who demonstrated its superiority over the traditional Shewhart chart in detecting small mean shifts. Rakitzis and Antzoulakos [11] developed RS charts for monitoring process variability, a critical aspect in industries where variability is tightly controlled. Teoh et al. [12] introduced the RS CV chart, which serves as a superior alternative to the CV chart. Lim et al. [13] extended this work to the multivariate process by developing the RS MCV chart, where the developed chart surpasses the MCV chart. Goh et al. [14] developed a RS control chart specifically tailored for the gamma distribution. Teoh et al. [15] employed an optimal RS control chart based on the median run length to resolve the issue of skewness in the run length distribution, thereby improving the practical effectiveness of the RS control chart.

The VSI charts adjust the sampling interval based on the current state of the process, offering enhanced flexibility and cost efficiency in process monitoring. The earliest contribution to the VSI concept was made by [16], where the VSI $\overset{―}{X}$ chart was developed and shown to be superior to the fixed sampling interval (FSI) $\overset{―}{X}$ chart. Saccucci et al. [17] integrated the VSI approach with the EWMA chart to develop the VSI EWMA chart that provides a quicker detection of small shifts. Baxley, Costa [18,19] analyzed the benefits of using the VSI charts. Due to the inevitability of measurement errors in practical applications, Hu et al. [20] studied the performance of the VSI$\;\overset{―}{X}$ control chart in the presence of measurement errors and provided practical applications. The application of VSI in CV monitoring was first explored by [21] who introduced the VSI EWMA CV chart and demonstrated its effectiveness in detecting small shifts in the CV. Ng et al. [22] suggested the VSI EWMA *t* chart with auxiliary information and evaluated its robustness towards estimation error. Chew et al. [23] introduced the VSI RS $\overset{―}{X}$ char*t* and found that the chart beats the RS $\overset{―}{X}$ chart. Yeong et al. [24] proposed the VSI RS CV chart which performs better than the RS CV chart. Yeong et al. [25] presented the RS CV chart with both the variable sample size and VSI features to further enhance the performance of the RS CV chart. Antzoulakos et al. [26] proposed a RS Max chart based on both the variable sample size and VSI features that adjusts the sample size and sampling interval according to the current cumulative score in making the chart more sensitive to process shifts.

In recent years, an increasing number of researchers have studied MCV control charts, as their applications have become more widespread in industries, such as in semiconductor manufacturing, aerospace, finance and hospital service quality monitoring. Although investigations on the RS charts for monitoring the process CV and MCV have been made in the literature and that the VSI strategy has been integrated into various types of control charts, the fusion of the RS and VSI methods for monitoring the process MCV remains unexplored. This study aims to fill this gap by developing a novel approach that integrates the benefits of the RS statistic and VSI technique to monitor the process MCV. In this paper, the VSI RS chart for monitoring the MCV is proposed. The main objective of proposing this new chart is to reduce the time needed to detect shifts in the process MCV, hence, contributing to the advancement of theoretical developments of new SPC methods and practical applications in industry. Henceforth, the organization of this paper is as follows. Section 2 reviews the MCV chart, while Section 3 presents the proposed VSI RS MCV chart. Optimal designs of the proposed VSI RS MCV chart in minimizing the out-of-control average time to signal (ATS) and expected average time to signal (EATS) values are presented in Section 4. Section 5 compares the performances of the proposed VSI RS MCV with existing MCV and RS MCV charts, in terms of the ATS, EATS and standard deviation of the time to signal (SDTS) criteria. Real life data are used to illustrate the application of the proposed VSI RS MCV chart in Section 6. Finally, conclusions are drawn and future research suggestions are made in Section 7.

## 2. A review of the MCV chart

Suppose that a *v* variate random sample of size *n* exists and is given by $X_{\text{1}},X_{\text{2}},\ldots ,X_{n}$, where v is the number of quality characteristics monitored simultaneously. These *v* variate observations $X_{i}$, for *i* = 1, 2, …, *n*, are independent and identically distributed having the *v* variate normal distribution, i.e., $X_{i}\sim N_{v}(\mu ,\Sigma )$, where μ and Σ are the mean vector and covariance matrix, respectively. Note that $X_{i}={\left(X_{i\text{1}},X_{i\text{2}},\ldots ,X_{iv}\right)}^{T}$ and $\mu ={\left(\mu_{\text{1}},\mu_{\text{2}},\ldots ,\mu_{v}\right)}^{T}.$

According to [27], the MCV statistic is defined as


$$\gamma ={\left(\mu^{T}\Sigma^{-\text{1}}\mu \right)}^{-\frac{\text{1}}{\text{2}}}.$$ (1)

The sample MCV, $\hat{\gamma }$ can be computed as


$$\hat{\gamma }={\left({\overset{―}{X}}^{T}S^{-\text{1}}\overset{―}{X}\right)}^{-\frac{\text{1}}{\text{2}}},$$ (2)

where the sample mean vector $\overset{―}{X}$ and sample covariance matrix S are computed as


$$\begin{array}{c} \overset{―}{X}={\left(\frac{\text{1}}{n}\sum_{i=\text{1}}^{n}X_{i\text{1}}\;,\frac{\text{1}}{n}\sum_{i=\text{1}}^{n}X_{i\text{2}}\;,\ldots ,\frac{\text{1}}{n}\sum_{i=\text{1}}^{n}\;{\text{X}}_{iv}\right)}^{T} \end{array}$$ (3)

and


$$S=\frac{\text{1}}{n-\text{1}}\sum_{i=\text{1}}^{n}\left(X_{i}-\overset{―}{X}\right){\left(X_{i}-\overset{―}{X}\right)}^{T}.$$ (4)

According to [28] and [3], the cumulative distribution function (cdf) of $\hat{\gamma }$ can be derived as


$$F_{\hat{\gamma }}(y|n,\;v,\;\delta )=\text{1}-F_{F}\left(\;\frac{n(n-v)}{(n-\text{1})vy^{\text{2}}}|\;v,n-v,\delta \right),$$ (5)

where $F_{F}(\;\cdot \;|v,\;n-v,\;\delta )$ is the cdf of the non-central F-distribution with v and n−v degrees of freedom and non-centrality parameter $\delta =n/{\left(\tau \gamma_{\text{0}}\right)}^{\text{2}}$.

For the upward MCV chart, the upper control limit (UCL) is


$$\text{UCL}=F_{\hat{\gamma }}^{-\text{1}}\left(\text{1}-\alpha |n,v,\delta_{\text{0}}\right)=\sqrt{\frac{n(n-v)}{(n-\text{1})v}\left[\frac{\text{1}}{F_{F}^{-\text{1}}\left(\alpha |v,n-v,\delta_{\text{0}}\right)}\right]}$$ (6)

and for the downward MCV chart, the lower control limit (LCL) is


$$\text{LCL}={\text{F}}_{\hat{\gamma }}^{-\text{1}}\left(\alpha |n,v,\delta_{\text{0}}\right)=\sqrt{\frac{n(n-v)}{(n-\text{1})v}\left[\frac{\text{1}}{F_{F}^{-\text{1}}\left(\text{1}-\alpha |v,n-v,\delta_{\text{0}}\right)}\right]},$$ (7)

where $\alpha =\text{1}/\text{AR}{\text{L}}_{\text{0}}$ and ${\text{ARL}}_{\text{0}}$ represents the desired in-control average run length (ARL) value specified by the practitioner. Note that $F_{F}^{-\text{1}}\left(\cdot |v,n-v,\delta_{\text{0}}\right)$ in Equations (6) and (7) is the inverse cdf of the non-central *F* distribution with v and n−v degrees of freedom, while $\delta_{\text{0}\;}\left(=\;n/\gamma_{\text{0}}^{\text{2}}\right)$ is obtained from *δ* by letting *τ* = 1. The out-of-control MCV is $\gamma_{\text{1}}=$ $\tau \gamma_{\text{0}}$, where τ≠1. When τ=1, the process MCV is in-control and $\gamma_{\text{0}}$ represents the in-control MCV.

ARL is a suitable measure in evaluating the performance of control charts with FSI. A VSI chart, on the other hand, uses variable sampling intervals, where the sampling interval between any two sampling points is different. Therefore, the ATS is a more suitable measure than the ARL in evaluating the average time required in detecting a process shift. To facilitate comparison between VSI and FSI charts, all the charts are compared using the ATS criterion in this paper. Let P denote the probability that a process is considered as out-of-control. According to [3], for the upward MCV chart,


$$P=\text{P}\left(\hat{\gamma }>\text{UCL}\right)=\text{1}-{\text{F}}_{\hat{\gamma }}\left(\text{UCL}|n,n-v,\delta_{\text{1}}\right),$$ (8)

while for the downward MCV chart,


$$P=\text{P}\left(\hat{\gamma }<\text{LCL}\right)={\text{F}}_{\hat{\gamma }}\left(\text{LCL}|n,n-v,\delta_{\text{1}}\right).$$ (9)

The ATS and SDTS for both the upward and downward MCV charts are computed as


$$\text{ATS}(\tau )=\text{ARL}\times \text{FSI}=\frac{\text{1}}{P}\times \text{FSI}$$ (10)

and


$$\text{SDTS}(\tau )=\text{SDRL}\times \text{FSI}=\frac{\sqrt{\text{1}-P}}{P}\times \text{FSI},$$ (11)

where *P* is defined for the upward and downward MCV charts in Equations (8) and (9), respectively.

## 3. The proposed VSI RS MCV charts

This study presents two VSI RS MCV charts, namely the upward and downward variants. In the design of the upward and downward RS MCV charts, Lim et al. [13] adopted


$${\text{LCL}}_{\text{0}}=\text{UC}{\text{L}}_{\text{0}}=F_{\gamma }^{-\text{1}}\left(\text{0}.\text{5}|n,v,\delta_{\text{0}}\right).$$ (12)

**Fig 1:**
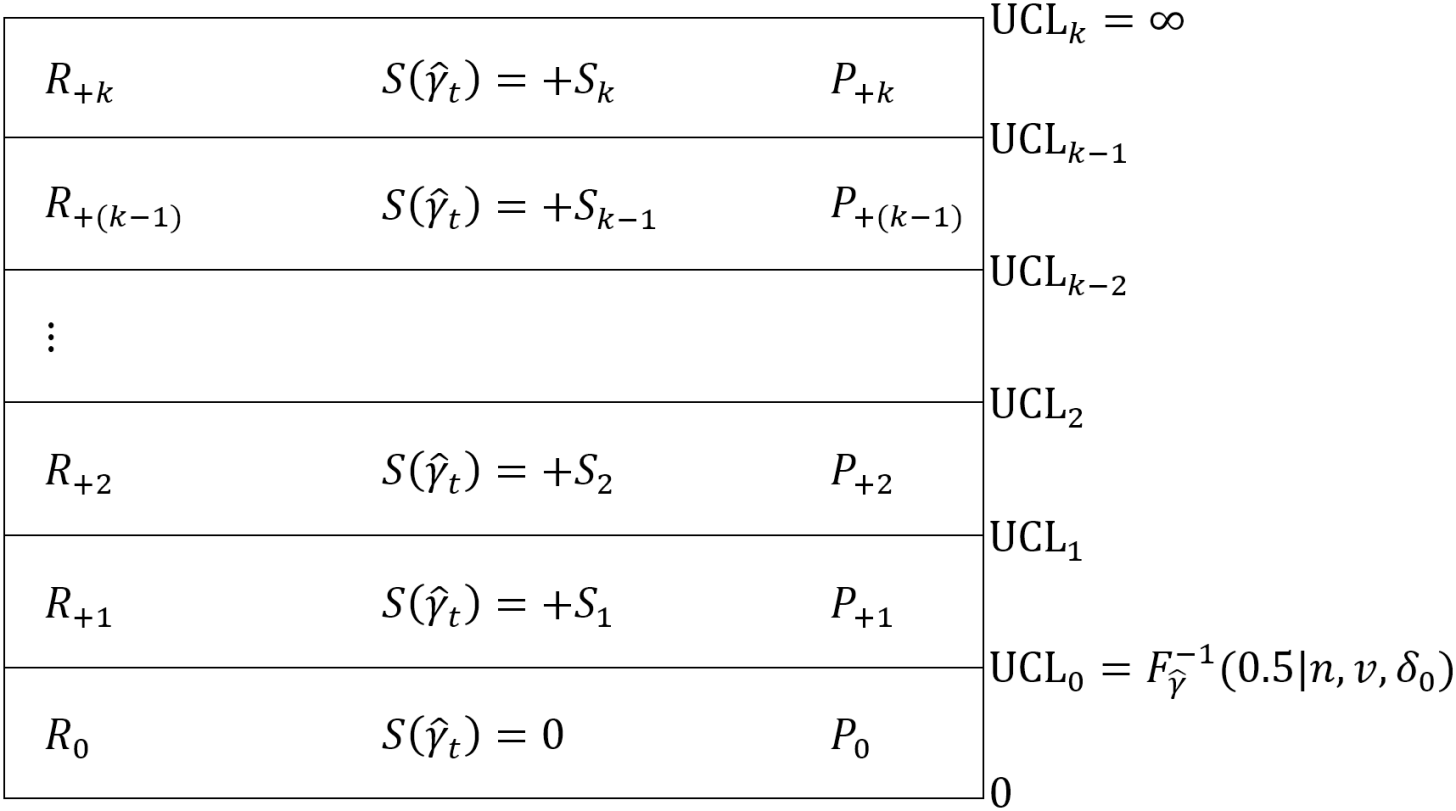
*k* regions upward VSI RS MCV chart with its corresponding scores and probabilities.

**Fig 2:**
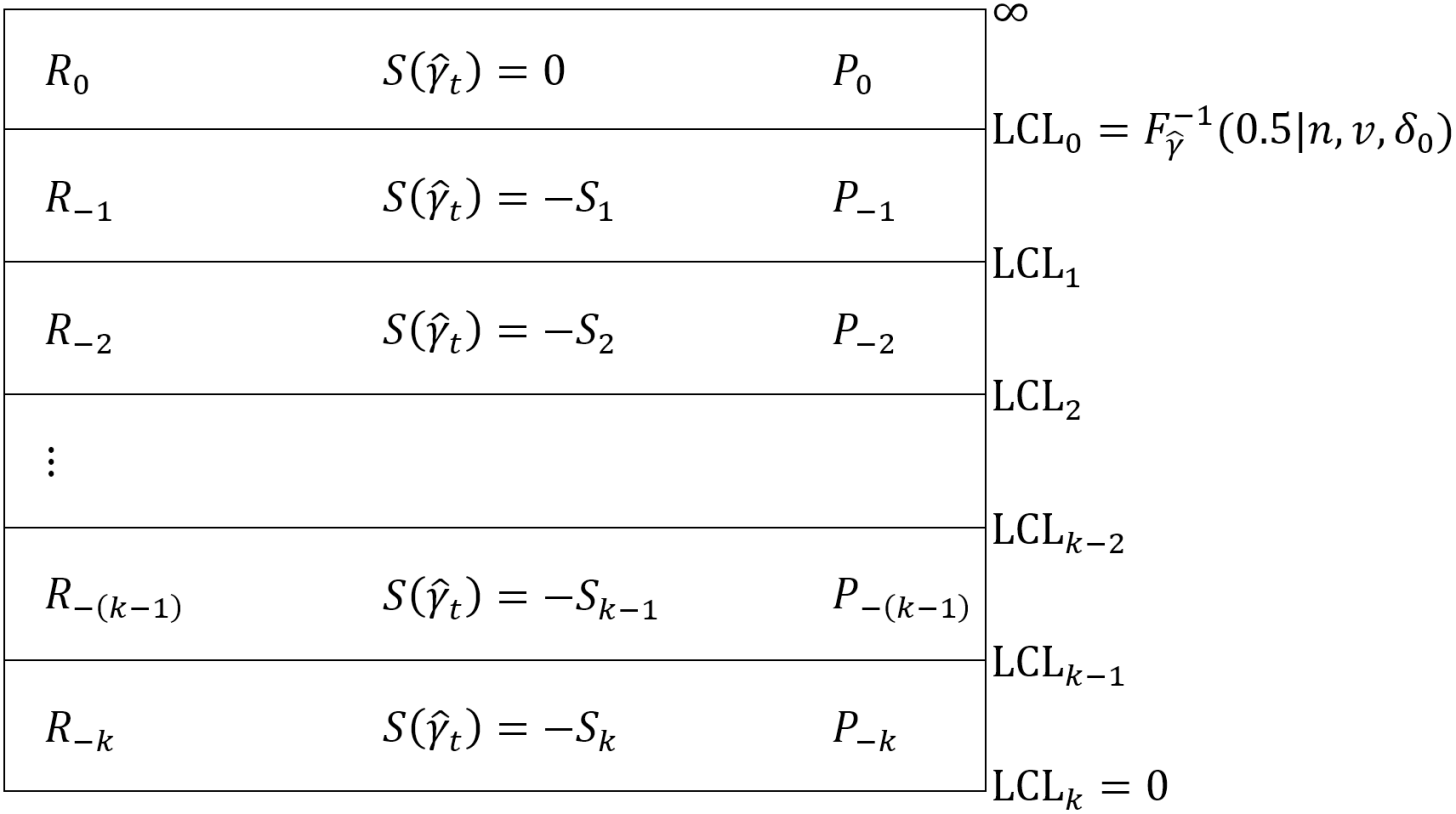
*k* regions downward VSI RS MCV chart with its corresponding scores and probabilities.

Figs 1 and 2 depict the *k*-regions upward and downward VSI RS MCV charts with their corresponding scores and probabilities, respectively.

For the upward VSI RS MCV chart in Fig 1, the control limits of the *k*-regions above $\text{UC}{\text{L}}_{\text{0}}$ satisfy the constraint $\text{0}<{\text{UCL}}_{\text{0}}<{\text{UCL}}_{\text{1}}<\ldots <{\text{UCL}}_{k-\text{1}}<{\text{UCL}}_{k}$, where ${\text{UCL}}_{k}=\infty$. Each region above $\text{UC}{\text{L}}_{\text{0}}$ of the upward VSI RS MCV chart is assigned an integer score $S_{j}$, given by


$$\;S\left({\hat{\gamma }}_{t}\right)=S_{j},\text{ if }{\hat{\gamma }}_{t}\in ({\text{UCL}}_{j-\text{1}},{\text{UCL}}_{j}),\text{for }j=\text{1},\;\text{2},\ldots ,k\text{ and }t=\text{1},\;\text{2},\ldots ,$$ (13)

where $\text{0}\leq S_{\text{1}}\leq S_{\text{2}}\leq \cdots \leq S_{k}$ and *t* is the sample number.

The downward VSI RS MCV chart in Fig 2 is also composed of *k* regions that are separated by *k* lower control limits below the ${\text{LCL}}_{\text{0}}$. These lower control limits satisfy the constraint ${\text{LCL}}_{k}<{\text{LCL}}_{k-\text{1}}<\cdots <{\text{LCL}}_{\text{1}}<{\text{LCL}}_{\text{0}}<\infty$, where ${\text{LCL}}_{k}=\text{0}$ and each region is assigned the score ${-S}_{j}$, given as


$$S\left({\hat{\gamma }}_{t}\right)=-S_{j},\text{ if }{\hat{\gamma }}_{t}\in {(\text{LCL}}_{j},{\text{LCL}}_{j-\text{1}}),\text{ for }j=\text{1},\text{2},\ldots ,k\text{ and }t=\text{1},\text{2},\ldots ,$$ (14)

where $-S_{k}\leq \cdots \leq -S_{\text{2}}\leq -S_{\text{1}}\leq \text{0}$.

Let $U_{t}$ and $L_{t}$ represent the upper and lower cumulative scores for the upward and downward VSI RS MCV charts, respectively, as defined in [13].


$$U_{t}=\left\{ \begin{array}{cc} \;\;\;\;\;\;\;\;\;\;\;\;\;\;\;\;\;\;\;\;\;\;\;\;\;\;\;\;\;\;\text{0}, & \text{ if }{\hat{\gamma }}_{t}<\text{UC}{\text{L}}_{\text{0}}\\ U_{t-\text{1}}+S\left({\hat{\gamma }}_{t}\right), & \text{ if }{\hat{\gamma }}_{t}\geq \text{UC}{\text{L}}_{\text{0}} \end{array}\right.\;$$ (15)

and


$$L_{t}=\left\{ \begin{array}{cc} \;\;\;\;\;\;\;\;\;\;\;\;\;\;\;\;\;\;\;\;\;\;\;\;\;\;\;\;\text{0}, & \;\;\;\;\text{ if }{\hat{\gamma }}_{t}>\text{LC}{\text{L}}_{\text{0}}\\ L_{t-\text{1}}+S\left({\hat{\gamma }}_{t}\right), & \text{ if }{\hat{\gamma }}_{t}\;\leq \text{ LC}{\text{L}}_{\text{0}} \end{array},\right.\;$$ (16)

where $U_{\text{0}}=\text{0}$ and $L_{\text{0}}=\text{0}$ are set as the initial values of the cumulative scores.

Let $T_{t}$ be the sampling interval for taking the next sample MCV, $\hat{\gamma }$, where the cumulative scores $U_{t}$ and $L_{t}$ determine the length of the sampling interval as follows:


$$T_{t}=\left\{ \begin{array}{cc} d_{\text{1}}, & \text{if }+\frac{S_{k}}{G}\leq U_{t}<+S_{k}\text{ or }-S_{k}<L_{t}\leq -\frac{S_{k}}{G}\mathrm{\backslash vspace}1mm\\ d_{\text{2}}, & \;\;\;\;\;\;\;\;\;\;\;\;\;\;\;\;\;\;\text{if }\text{0}\leq U_{t}<+\frac{S_{k}}{G}\text{ or }-\frac{S_{k}}{G}<L_{t}\leq \text{0} \end{array}.\right.\;$$ (17)

In Equation (17), *G* is a user-defined positive integer that regulates the threshold for transitioning between the two sampling intervals ($d_{\text{1}}$ and $d_{\text{2}}$), where $d_{\text{1}}$ represents the short sampling interval and $d_{\text{2}}$ denotes the long sampling interval, specifically, $\text{0}<d_{\text{1}}<d_{\text{2}}.\;$The proposed VSI RS MCV chart will signal an out-of-control when the cumulative score $U_{t}$ or $L_{t}$ equals or exceeds the triggering score $+S_{k}$ or $-S_{k}$, respectively.

For the upward VSI RS MCV chart, the upper control limits are computed as


$${\text{UCL}}_{j}=K\times {\text{F}}_{\hat{\gamma }}^{-\text{1}}\left({\text{1}-\alpha }_{j}|n,v,\delta_{\text{0}}\right),\;\text{for }j=\text{1},\text{2},\ldots ,k-\text{1}$$ (18)

and for the downward VSI RS MCV chart, the lower control limits are computed as


$${\text{LCL}}_{j}=K\times F_{\hat{\gamma }}^{-\text{1}}\left(\alpha_{j}|n,v,\delta_{\text{0}}\right),\text{ for }j=\text{1},\text{2},\ldots ,k-\text{1},$$ (19)

where K is a constant selected to obtain the specified ${\text{ARL}}_{\text{0}}$ performance. Also,


$$\alpha_{j}=\Phi \left(\frac{\text{3}j}{k-\text{1}}\right),\text{ for }j=\text{1},\text{2},\ldots ,k-\text{1},$$ (20)

where Φ( · ) is the standard normal cdf.

The upward and downward VSI RS MCV charts are constructed as follows:

Step 1. Set the upper $\left(U_{\text{0}}\right)$ and lower $\left(L_{\text{0}}\right)$ initial cumulative scores as zero. Determine the sample size (*n*), number of regions (k), switching threshold (G), in-control MCV ($\gamma_{\text{0}}$) and short sampling interval $\left(d_{\text{1}}\right)$. If $\gamma_{\text{0}}$ is unknown, use its estimate ${\hat{\gamma }}_{\text{0}}=\sqrt{\frac{\text{1}}{m}\sum_{t=\text{1}}^{m}{\hat{\gamma }}_{t}^{\text{2}}}$, where m denotes the number of in-control Phase-I samples, as described in [3].
Step 2. Calculate $\text{UC}{\text{L}}_{\text{0}}$ and $\text{LC}{\text{L}}_{\text{0}}$ using Equation (12).
Step 3. Calculate the upper control limits, $\text{UC}{\text{L}}_{j},\text{for }j=\text{1},\text{2},\ldots ,k$ with Equation (18) for the upward VSI RS MCV chart, and the lower control limits, $\text{LC}{\text{L}}_{j},\text{ for }j=\text{1},\text{2},\ldots ,k$ with Equation (19) for the downward VSI RS MCV chart.
Step 4. Plot the sample MCV, ${\hat{\gamma }}_{t}$, on the upward or downward VSI RS MCV chart and compute the value of $S\left({\hat{\gamma }}_{t}\right)$ using Equation (13) or (14), based on the region where ${\hat{\gamma }}_{t}$ falls into.
Step 5. If ${\hat{\gamma }}_{t}\in R_{+j}$, for j=1,2,…,k, compute the upper cumulative score $U_{t}$ using Equation (15). If ${\hat{\gamma }}_{t}\in R_{-j}$, for j=1,2,…,k, compute the lower cumulative score $L_{t}$ using Equation (16). $U_{t}$ and $L_{t}$ are reset to zero if ${\hat{\gamma }}_{t}<{\text{UCL}}_{\text{0}}$ or ${\hat{\gamma }}_{t}>{\text{LCL}}_{\text{0}}$, respectively.
Step 6. If $+\frac{S_{k}}{G}\leq U_{t}<+S_{k}$ or $-S_{k}<L_{t}<-\frac{S_{k}}{G},$ the short sampling interval $d_{\text{1}}$ will be employed to take the next sample. Otherwise, the next sample will be taken after the long sampling interval, $d_{\text{2}}$, if $\text{0}\leq U_{t}<+\frac{S_{k}}{G}$ or $-\frac{S_{k}}{G}<L_{t}<\text{0}$.
Step 7. The VSI RS MCV chart will issue an out-of-control signal at sample t if the cumulative score $U_{t}\geq +S_{k}$ for the upward chart or $L_{t}\leq -S_{k}$ for the downward chart, hence, go to Step 8. Otherwise, return to Step 4.
Step 8. Identify and remove the assignable causes so that the process returns to the in-control condition again. Then return to Step 4.

For the upward VSI RS MCV chart, let $P_{+j}$ denote the probability of ${\hat{\gamma }}_{t}$ falling in the interval $\left({\text{UCL}}_{j-\text{1}},{\text{UCL}}_{j}\right)$, for j=1,2,…,k. Then $P_{+j}$ is computed as


$$\begin{array}{cc} {\;P}_{+j} & =\text{Pr}\left({\hat{\gamma }}_{t}\in ({\text{UCL}}_{j-\text{1}},{\text{UCL}}_{j}\right)\\ & =\text{Pr}\left({\hat{\gamma }}_{t}<{\text{UCL}}_{j}\right)-\text{Pr}\left({\hat{\gamma }}_{t}<{\text{UCL}}_{j-\text{1}}\right)\\ & =\text{1}-F_{F}\left(\;\frac{n(n-v)}{(n-\text{1})v{\text{UCL}}_{j}^{\text{2}}}|\;v,n-v,\delta \right)-\text{1}+F_{F}\left(\;\frac{n(n-v)}{(n-\text{1})v{\text{UCL}}_{j-\text{1}}^{\text{2}}}|\;v,n-v,\delta \right)\;\\ & =F_{F}\left(\;\frac{n(n-v)}{(n-\text{1})v{\text{UCL}}_{j-\text{1}}^{\text{2}}}|\;v,n-v,\delta \right)-F_{F}\left(\;\frac{n(n-v)}{(n-\text{1})v{\text{UCL}}_{j}^{\text{2}}}|\;v,n-v,\delta \right). \end{array}$$ (21)

Let $P_{\text{0}}$ denote the probability that ${\hat{\gamma }}_{t}$ falls below ${\text{UCL}}_{\text{0}}$. Then $P_{\text{0}}$ is computed as [13]


$${\;P}_{\text{0}}=\left\{ \begin{array}{cc} \;\;\;\;\;\;\;\;\;\;\;\;\;\;\;\;\;\;\;\;\;\;\;\;\;\;\;\;\;\;\;\;\;\;\;\;\;\;\;\;\;\;\;\;\;\;\;\;\;\;\;\;\;\;\;\;\;\;\;\;\;\;\;\;\;\;\;\;\;\;\;\;\text{0}.\text{5}, & \text{if }\tau =\text{1}\\ \text{1}-F_{F}\left(\;\frac{n(n-v)}{(n-\text{1})v{UCL}_{\text{0}}^{\text{2}}}|\;v,n-v,\delta \right), & \text{if }\tau \neq \text{1} \end{array}.\right.\;$$ (22)

In addition, for the downward VSI RS MCV chart, let $P_{-j}$ be the probability of ${\hat{\gamma }}_{t}$ falling in the interval $\left({\text{LCL}}_{j},{\text{LCL}}_{j-\text{1}}\right)$, for j=1,2,…,k. Then $P_{-j}$ is computed as [13]


$$P_{-j}=\text{Pr}\left({\hat{\gamma }}_{t}\in {\text{LCL}}_{j},{\text{LCL}}_{j-\text{1}}\right)$$

$$\begin{array}{cc} & \;=\text{Pr}\left({\hat{\gamma }}_{t}<{\text{LCL}}_{j-\text{1}}\right)-\text{Pr}\left({\hat{\gamma }}_{t}<{\text{LCL}}_{j}\right)\\ & \;=\text{1}-F_{F}\left(\;\frac{n(n-v)}{(n-\text{1})v{\text{LCL}}_{j-\text{1}}^{\text{2}}}|\;v,n-v,\delta \right)-\text{1}+F_{F}\left(\;\frac{n(n-v)}{(n-\text{1})v{\text{LCL}}_{j}^{\text{2}}}|\;v,n-v,\delta \right)\\ & \;=F_{F}\left(\;\frac{n(n-v)}{(n-\text{1})v{\text{LCL}}_{j}^{\text{2}}}|\;v,n-v,\delta \right)-F_{F}\left(\;\frac{n(n-v)}{(n-\text{1})v{\text{LCL}}_{j-\text{1}}^{\text{2}}}|\;v,n-v,\delta \right). \end{array}$$ (23)

Also, let $P_{\text{0}}$ be the probability of ${\hat{\gamma }}_{t}$ falling above ${\text{LCL}}_{\text{0}}$. Then $P_{\text{0}}$ is computed as [13]


$$P_{\text{0}}=\left\{ \begin{array}{cc} \;\;\;\;\;\;\;\;\;\;\;\;\;\;\;\;\;\;\;\;\;\;\;\;\;\;\;\;\;\;\;\;\;\;\;\;\;\;\;\;\;\;\;\;\;\;\;\;\;\;\;\;\;\;\;\;\;\;\;\;\;\text{0}.\text{5}, & \text{if }\tau =\text{1}\\ F_{F}\left(\;\frac{n(n-v)}{(n-\text{1})v\text{LC}{\text{L}}_{\text{0}}^{\text{2}}}|\;v,n-v,\delta \right), & \text{if }\tau \neq \text{1} \end{array}\right.\;.$$ (24)

According to [29], the steady state ATS and SDTS of the VSI RS MCV chart are computed using the following formulae:


$$\text{ATS}(\tau )=q^{T}\alpha_{\text{1}}-q^{T}t$$ (25)

and


$$\text{SDTS}(\tau )=\sqrt{q^{T}\alpha_{\text{2}}-{\left(q^{T}\alpha_{\text{1}}\right)}^{\text{2}}},$$ (26)

where $\alpha_{\text{1}}=(I-Q{)}^{-\text{1}}t$ and $\alpha_{\text{2}}=(I-Q{)}^{-\text{1}}\left(\text{2}D_{t}\alpha_{\text{1}}-t^{\left(\text{2}\right)}\right)$. Here, Q is the transition probability matrix (TPM) for the transient states of size m×m. The construction of the TPM for the VSI RS MCV chart can be made using the approach presented in [13]. Note that I is the identity matrix with dimension $m\times m,\;t={\left(T_{\text{1}},T_{\text{2}},\ldots ,T_{m}\right)}^{\prime }$ is the vector of sampling intervals with dimension m × 1, where $T_{t}=d_{\text{1}}$ or $d_{\text{2}}$, determined based on Equation (17), for t=1, 2,…,m, and $q^{T}$ is the steady-state probability vector. For obtaining ***q***, first compute vector ***s*** by solving $s={\left(Q^{*}\right)}^{T}s$, based on the constraint $1^{T}s=\text{1}$, where $Q^{*}$ represents the TPM with the absorbing state when the process is in-control and 1 is a vector having ones in all of its entries. Then compute the vector $s^{*}$ from the vector s by discarding the entry in the vector s that corresponds to the absorbing state. Next, compute q as $q={\left(1^{T}s^{*}\right)}^{-\text{1}}s^{*}$, based on the approach presented in [30]. Additionally, note that the matrix $D_{t}$ is a diagonal matrix whose diagonal elements are taken from the vector t.

In order to ensure a fair comparison with other control charts, the in-control average sampling interval $\left(\text{AS}{\text{I}}_{\text{0}}\right)$ for all competing charts has to be equal. The formula for computing $\text{AS}{\text{I}}_{\text{0}}$ of the VSI RS MCV chart is given in [17]:


$$\text{AS}{\text{I}}_{\text{0}}=d_{\text{1}}P+d_{\text{2}}(\text{1}-P),$$ (27)

where P is the proportion of time for adopting the short sampling interval $\left(d_{\text{1}}\right)$, while 1−P is the proportion of time for using the long sampling interval $\left(d_{\text{2}}\right)$.

The performance of the proposed VSI RS MCV chart is evaluated using the ATS criterion when the exact shift size can be specified. However, in practical situations, pinpointing the exact shift size can be challenging. To address this challenge, the EATS criterion is employed to assess the chart’s performance when process shifts occur within the range defined by the minimum and maximum shift sizes. According to [29], the EATS value for the VSI RS MCV chart over the shift interval ${(\tau }_{\text{min}},\tau_{\text{max}})$ can be calculated as follows:


$$\text{EATS}\left(\tau_{\text{min}},\tau_{\text{max}}\right)=\frac{\text{1}}{\tau_{\text{max}}-\tau_{\text{min}}}\int_{\tau_{\text{min}}}^{\tau_{\text{max}}}\;\text{ATS}(\tau )d\tau .$$ (28)

## 4. Optimal designs of the VSI RS MCV chart

To compute the optimal parameters $d_{\text{2}}$ and *K*, as well as the optimal scores $\left\{\pm S_{\text{1}},\pm S_{\text{2}},\ldots ,\pm S_{k}\right\}$, the optimization model for minimizing the steady state out-of-control ATS (ATS(τ)) (*τ* ≠ 1) value in (29a − c) and (30a − c) for the upward and downward VSI RS MCV charts, respectively, are employed.


$$\begin{array}{cc} \; & \text{ Minimize }\text{ATS}(\tau )\\ \; & d_{\text{2}},K,\left\{S_{\text{1}},S_{\text{2}},\ldots ,S_{\text{k}}\right\} \end{array},$$ (29a)

subject to the constraints


$$\text{ATS}(\text{1})=\text{AT}{\text{S}}_{\text{0}\;}\;$$ (29b)

and


$$\text{AS}{\text{I}}_{\text{0}}=d_{\text{0}},$$ (29c)

or


$$\begin{array}{c} \text{ Minimize }\text{ATS}(\tau )\\ d_{\text{2}},K,\left\{-S_{\text{1}},-S_{\text{2}},\ldots ,-S_{\text{k}}\right\} \end{array},$$ (30a)

subject to the constraints


$$\text{ATS}(\text{1})=\text{AT}{\text{S}}_{\text{0}}$$ (30b)

and


$$\text{AS}{\text{I}}_{\text{0}}=d_{\text{0}}.$$ (30c)

In Equations (29b) and (30b), ${\text{ATS}}_{\text{0}}$ is the desired ATS(1) value, while in Equations (29c) and (30c), $d_{\text{0}}$ is the desired $\text{AS}{\text{I}}_{\text{0}}$ value. In this study, we adopt the approach suggested by [31] to set the $d_{\text{1}}$ value first, followed by obtaining the corresponding $d_{\text{2}}$ value using the above-mentioned optimization model.

The steps in finding the optimal parameter and score combination for the VSI RS MCV charts in minimizing ATS(τ) are as follows:

Step 1. Specify the values of ${\text{ATS}}_{\text{0}},d_{\text{0}},\;v,n,\tau \;(\neq \;\text{1}),G,d_{\text{1}}$ and $\gamma_{\text{0}}$. Then, initialize ${\text{ATS}}_{\text{min}}=\infty$.
Step 2. Select a score combination $\left\{S_{\text{1}},S_{\text{2}},\ldots ,S_{k}\right\}$ which satisfies the constraint $\text{0}\leq S_{\text{1}}\leq S_{\text{2}}\leq \cdots \leq S_{k}\leq \text{10}$ for the upward chart. In the case of the downward chart, select a score combination $\left\{-S_{\text{1}},-S_{\text{2}},\ldots ,-S_{k}\right\}$ which satisfies the constraint $-\text{10}\leq -S_{k}\leq \cdots \leq -S_{\text{2}}\leq -S_{\text{1}}\leq \text{0}$. If no new combination is possible, proceed to Step 6.
Step 3. Compute the values of $d_{\text{2}}$ and *K* based on the score combination selected in step 2 that satisfy (29a − c) for the upward chart and (30a − c) for the downward chart.
Step 4. Compute ATS(τ) using Equation (25) for the upward and downward charts, for the shift size τ, based on the parameters and scores from Steps 2 and 3.
Step 5. If $\text{ATS}(\tau )<{\text{ATS}}_{\text{min}}$, let ${\text{ATS}}_{\text{min}}=\text{ATS}(\tau )$ and keep the $d_{\text{2}}$ and *K* values, as well as scores obtained from steps 2–3 as the corresponding optimal parameters. Then, return to Step 2.
Step 6. $\text{AT}{\text{S}}_{\text{min}}$ is taken as the minimum ATS(τ) value and the corresponding $\left(d_{\text{2}},K,\left\{S_{\text{1}},S_{\text{2}},\ldots ,S_{k}\right\}\right)$ or $\left(d_{\text{2}},K,\left\{-S_{\text{1}},-S_{\text{2}},\ldots ,-S_{k}\right\}\right)$ is used as the optimal parameter and score combination of the upward or downward VSI RS MCV chart, respectively, for the ${\text{ATS}}_{\text{0}},d_{\text{0}},v,n,\tau \;(\neq \;\text{1}),G,d_{\text{1}}$ and $\gamma_{\text{0}}$ values specified in Step 1.

The optimal parameter and score combination that minimizes the steady state EATS$\left(\tau_{\text{min}},\tau_{\text{max}}\right)$ value for the shift interval $\left(\tau_{\text{min}},\tau_{\text{max}}\right)$ can also be obtained by using the above-mentioned Steps 1–6 but with some changes. Firstly, the exact shift size *τ* (≠ 1) specified in Step 1 is substituted with the specified shift interval $\left(\tau_{\text{min}},\tau_{\text{max}}\right)$, where $\tau_{\text{min}}$ <$\tau_{\text{max}}$. Secondly, ATS(τ) in Steps 4–6 is replaced by EATS$\left(\tau_{\text{min}},\tau_{\text{max}}\right)$, while ${\text{ATS}}_{\text{min}}$ in Steps 5 and 6 is replaced by $\text{E}{\text{ATS}}_{\text{min}}$. Also, in Step 4, Equation (25) is replaced by Equation (28). Furthermore, the in-control EATS value computed using Equation (28) replaces ATS (1) in Equations (29b) and (30b).

To obtain the optimal parameter and score combination $\left(d_{\text{2}},K,\left\{S_{\text{1}},S_{\text{2}},\ldots ,S_{k}\right\}\right)$ or $\left(d_{\text{2}},K,\left\{-S_{\text{1}},-S_{\text{2}},\ldots ,-S_{k}\right\}\right)$ that minimizes the ATS(τ) (*τ* ≠ 1) (or $\text{EATS}\left(\tau_{\text{min}},\tau_{\text{max}}\right))$ value, optimization programs in MATLAB are developed. The input parameters specified are${\text{ATS}}_{\text{0}}\in \{\text{2}\text{0}\text{0},\;\text{5}\text{0}\text{0}\}$, v=2,  n∈{5, 10}, G∈{3, 4}, $d_{\text{1}}\in \{\text{0}.\text{0}\text{5},\;\text{0}.\text{1}\}$, $\gamma_{\text{0}}\in \{\text{0}.\text{1}\text{0},\;\text{0}.\text{3}\text{0},\;\text{0}.\text{5}\text{0}\}$and k=4. The four regions upward and downward VSI RS MCV charts are considered. The shift sizes, τ∈{1.05, 1.10, 1.25, 1.5} and {0.50, 0.60, 0.75, 0.90} are considered for the upward and downward VSI RS MCV charts, respectively, when the charts are designed to minimize ATS(τ) (*τ* ≠ 1). In the case of minimizing EATS$\left(\tau_{\text{min}},\tau_{\text{max}}\right)$, the shift intervals used are $\left(\tau_{\text{min}},\tau_{\text{max}}\right)$ = (1, 2) and (0.5, 1) for the upward and downward VSI RS MCV charts, respectively.

The optimal parameter and score combination in minimizing ATS(τ) (*τ* ≠ 1), for $\text{AT}{\text{S}}_{\text{0}}=\text{200}$ are given in Tables 1 and 2 for the upward and downward charts, respectively, while Tables 3 and 4 provide the said optimal parameter and score combinations for the upward and

**Table 1:** Optimal parameters of the upward VSI RS MCV chart in minimizing ATS(*τ*) when $\text{AT}{\text{S}}_{\text{0}}$ = 200 and *v* = 2.

*G*	$d_{1}$	*τ*	$\gamma_{0}$	*n* = 5						*n* = 10					
				$d_{2}$	*K*	$S_{1}$	$S_{2}$	$S_{3}$	$S_{4}$	$d_{2}$	*K*	$S_{1}$	$S_{2}$	$S_{3}$	$S_{4}$
3	0.05	1.05	0.1	1.1915	1.0935	0	1	2	3	1.1885	1.0619	0	1	2	3
			0.3	1.1878	1.1068	0	2	3	5	1.2350	1.0368	0	2	3	6
			0.5	1.1796	1.1384	0	1	2	3	1.2322	1.0451	0	2	3	6
		1.1	0.1	1.1915	1.0935	0	2	3	5	1.1885	1.0619	0	2	3	5
			0.3	1.1878	1.1068	0	2	3	5	1.1861	1.0700	0	2	5	6
			0.5	1.1796	1.1384	0	1	2	3	1.2322	1.0451	0	2	3	6
		1.25	0.1	1.1915	1.0935	0	2	3	5	1.1885	1.0619	0	1	2	3
			0.3	1.1878	1.1068	0	2	3	5	1.2350	1.0368	0	2	3	6
			0.5	1.1796	1.1384	0	2	3	5	1.2322	1.0451	0	2	3	6
		1.5	0.1	1.1915	1.0935	0	2	3	5	1.2362	1.0328	0	2	3	6
			0.3	1.1878	1.1068	0	2	3	5	1.2350	1.0368	0	2	3	6
			0.5	1.1796	1.1384	0	2	3	5	1.2322	1.0451	0	2	3	6
	0.1	1.05	0.1	1.1817	1.0935	0	1	2	3	1.1790	1.0619	0	1	2	3
			0.3	1.1782	1.1068	0	2	3	5	1.2230	1.0368	0	2	3	6
			0.5	1.1705	1.1384	0	1	2	3	1.2204	1.0451	0	2	3	6
		1.1	0.1	1.1817	1.0935	0	2	3	5	1.1790	1.0619	0	2	3	5
			0.3	1.1782	1.1068	0	2	3	5	1.1766	1.0700	0	2	5	6
			0.5	1.1705	1.1384	0	1	2	3	1.2204	1.0451	0	2	3	6
		1.25	0.1	1.1817	1.0935	0	2	3	5	1.1790	1.0619	0	1	2	3
			0.3	1.1782	1.1068	0	2	3	5	1.1766	1.0700	0	2	5	6
			0.5	1.1705	1.1384	0	2	3	5	1.2204	1.0451	0	2	3	6
		1.5	0.1	1.1817	1.0935	0	2	3	5	1.2241	1.0328	0	2	3	6
			0.3	1.1782	1.1068	0	1	2	3	1.2230	1.0368	0	2	3	6
			0.5	1.1705	1.1384	0	2	3	5	1.2204	1.0451	0	2	3	6
4	0.05	1.05	0.1	1.2924	1.0046	0	2	5	8	1.2921	1.0031	0	3	5	10
			0.3	1.2916	1.0054	0	2	5	8	1.2919	1.0036	0	1	2	4
			0.5	1.2911	1.0070	0	3	5	10	1.2918	1.0045	0	1	2	4
		1.1	0.1	1.2924	1.0046	0	3	5	10	1.2921	1.0031	0	1	2	4
			0.3	1.2916	1.0054	0	2	5	8	1.2919	1.0036	0	1	2	4
			0.5	1.2911	1.0070	0	2	5	8	1.2918	1.0045	0	1	2	4
		1.25	0.1	1.2924	1.0046	0	1	2	4	1.2921	1.0031	0	2	5	8
			0.3	1.2916	1.0054	0	3	5	10	1.2919	1.0036	0	1	2	4
			0.5	1.2911	1.0070	0	1	2	4	1.2918	1.0045	0	1	2	4
		1.5	0.1	1.2924	1.0046	0	3	5	10	1.3457	0.9786	0	2	3	8
			0.3	1.2916	1.0054	0	1	2	4	1.3471	0.9757	0	2	3	8
			0.5	1.2911	1.0070	0	1	2	4	1.2918	1.0045	0	1	2	4
	0.1	1.05	0.1	1.2774	1.0046	0	2	5	8	1.2771	1.0031	0	3	5	10
			0.3	1.2765	1.0054	0	2	5	8	1.2769	1.0036	0	2	5	8
			0.5	1.2761	1.0070	0	3	5	10	1.2768	1.0045	0	1	2	4
		1.1	0.1	1.2774	1.0046	0	3	5	10	1.2771	1.0031	0	1	2	4
			0.3	1.2765	1.0054	0	2	5	8	1.2769	1.0036	0	2	5	8
			0.5	1.2761	1.0070	0	2	5	8	1.2768	1.0045	0	1	2	4
		1.25	0.1	1.2774	1.0046	0	1	2	4	1.2771	1.0031	0	2	5	8
			0.3	1.2765	1.0054	0	3	5	10	1.2769	1.0036	0	1	2	4
			0.5	1.2761	1.0070	0	2	5	8	1.2768	1.0045	0	1	2	4
		1.5	0.1	1.2774	1.0046	0	3	5	10	1.3278	0.9786	0	2	3	8
			0.3	1.2765	1.0054	0	3	5	10	1.2769	1.0036	0	3	5	10
			0.5	1.2761	1.0070	0	1	2	4	1.2768	1.0045	0	1	2	4

**Table 2:** Optimal parameters of the downward VSI RS MCV chart in minimizing ATS(*τ*) when $\text{AT}{\text{S}}_{\text{0}}$ = 200 and *v* = 2.

*G*	$d_{1}$	*τ*	$\gamma_{0}$	*n* = 5						*n* = 10					
				$d_{2}$	*K*	$S_{1}$	$S_{2}$	$S_{3}$	$S_{4}$	$d_{2}$	*K*	$S_{1}$	$S_{2}$	$S_{3}$	$S_{4}$
3	0.05	0.5	0.1	1.2339	0.9104	0	−1	−1	−3	1.2298	0.9502	0	−2	−3	−6
			0.3	1.2336	0.9095	0	−1	−1	−3	1.2296	0.9489	0	−2	−3	−6
			0.5	1.2340	0.9081	0	−1	−1	−3	1.2297	0.9468	0	−2	−3	−6
		0.6	0.1	1.2106	0.8782	−1	−2	−3	−9	1.2348	0.9545	0	−1	−1	−3
			0.3	1.2105	0.8773	−1	−2	−3	−9	1.2352	0.9533	0	−1	−1	−3
			0.5	1.2107	0.8759	−1	−2	−3	−9	1.2351	0.9513	0	−1	−1	−3
		0.75	0.1	1.2106	0.8782	−1	−2	−3	−9	1.2348	0.9545	0	−1	−1	−3
			0.3	1.2105	0.8773	−1	−2	−3	−9	1.2352	0.9533	0	−1	−1	−3
			0.5	1.2107	0.8759	−1	−2	−3	−9	1.2119	0.9366	−1	−2	−3	−9
		0.9	0.1	1.1436	0.4465	−1	−2	−2	−7	1.2115	0.9400	−1	−2	−3	−9
			0.3	1.1436	0.4445	−1	−2	−2	−7	1.2117	0.9387	−1	−2	−3	−9
			0.5	1.1436	0.4412	−1	−2	−2	−7	1.2119	0.9366	−1	−2	−3	−9
	0.1	0.5	0.1	1.2219	0.9104	0	−1	−1	−3	1.2181	0.9502	0	−2	−3	−6
			0.3	1.2216	0.9095	0	−1	−1	−3	1.2178	0.9489	0	−2	−3	−6
			0.5	1.2220	0.9081	0	−1	−1	−3	1.2179	0.9468	0	−2	−3	−6
		0.6	0.1	1.1999	0.8782	−1	−2	−3	−9	1.2181	0.9502	0	−2	−3	−6
			0.3	1.1998	0.8773	−1	−2	−3	−9	1.2231	0.9533	0	−1	−1	−3
			0.5	1.1999	0.8759	−1	−2	−3	−9	1.2230	0.9513	0	−1	−1	−3
		0.75	0.1	1.1999	0.8782	−1	−2	−3	−9	1.2227	0.9545	0	−1	−1	−3
			0.3	1.1998	0.8773	−1	−2	−3	−9	1.2231	0.9533	0	−1	−1	−3
			0.5	1.1999	0.8759	−1	−2	−3	−9	1.2011	0.9366	−1	−2	−3	−9
		0.9	0.1	1.1364	0.4465	−1	−2	−2	−7	1.2008	0.9400	−1	−2	−3	−9
			0.3	1.1364	0.4445	−1	−2	−2	−7	1.2008	0.9387	−1	−2	−3	−9
			0.5	1.1363	0.4412	−1	−2	−2	−7	1.2011	0.9366	−1	−2	−3	−9
4	0.05	0.5	0.1	1.3747	1.0945	0	−1	−1	−4	1.3568	1.0374	0	−2	−3	−8
			0.3	1.3742	1.0956	0	−1	−1	−4	1.3563	1.0383	0	−2	−3	−8
			0.5	1.3741	1.0975	0	−1	−1	−4	1.3563	1.0400	0	−2	−3	−8
		0.6	0.1	1.3747	1.0945	0	−1	−1	−4	1.3568	1.0374	0	−2	−3	−8
			0.3	1.3742	1.0956	0	−1	−1	−4	1.3563	1.0383	0	−2	−3	−8
			0.5	1.3811	0.7443	−1	−2	−2	−8	1.3563	1.0400	0	−2	−3	−8
		0.75	0.1	1.3810	0.7489	−1	−2	−2	−8	1.3568	1.0374	0	−2	−3	−8
			0.3	1.3810	0.7472	−1	−2	−2	−8	1.3563	1.0383	0	−2	−3	−8
			0.5	1.3811	0.7443	−1	−2	−2	−8	1.3563	1.0400	0	−2	−3	−8
		0.9	0.1	1.3296	0.4465	−1	−2	−2	−7	1.3741	0.8500	−1	−2	−3	−8
			0.3	1.3296	0.4445	−1	−2	−2	−7	1.3742	0.8470	−1	−2	−3	−8
			0.5	1.3296	0.4412	−1	−2	−2	−7	1.3746	0.8420	−1	−2	−3	−8
	0.1	0.5	0.1	1.3553	1.0945	0	−1	−1	−4	1.2756	0.9946	0	−1	−2	−4
			0.3	1.3548	1.0956	0	−1	−1	−4	1.2760	0.9945	0	−1	−2	−4
			0.5	1.3548	1.0975	0	−1	−1	−4	1.2759	0.9943	0	−1	−2	−4
		0.6	0.1	1.3553	1.0945	0	−1	−1	−4	1.3383	1.0374	0	−2	−3	−8
			0.3	1.3548	1.0956	0	−1	−1	−4	1.3379	1.0383	0	−2	−3	−8
			0.5	1.3613	0.7443	−1	−2	−2	−8	1.3379	1.0400	0	−2	−3	−8
		0.75	0.1	1.3612	0.7489	−1	−2	−2	−8	1.3383	1.0374	0	−2	−3	−8
			0.3	1.3613	0.7472	−1	−2	−2	−8	1.3379	1.0383	0	−2	−3	−8
			0.5	1.3613	0.7443	−1	−2	−2	−8	1.3379	1.0400	0	−2	−3	−8
		0.9	0.1	1.3127	0.4465	−1	−2	−2	−7	1.3548	0.8500	−1	−2	−3	−8
			0.3	1.3127	0.4445	−1	−2	−2	−7	1.3549	0.8470	−1	−2	−3	−8
			0.5	1.3126	0.4412	−1	−2	−2	−7	1.3553	0.8420	−1	−2	−3	−8

**Table 3:** Optimal parameters of the upward VSI RS MCV chart in minimizing ATS(*τ*) when $\text{AT}{\text{S}}_{\text{0}}$ = 500 and *v* = 2.

*G*	$d_{1}$	*τ*	$\gamma_{0}$	*n* = 5						*n* = 10					
				$d_{2}$	*K*	$S_{1}$	$S_{2}$	$S_{3}$	$S_{4}$	$d_{2}$	*K*	$S_{1}$	$S_{2}$	$S_{3}$	$S_{4}$
3	0.05	1.05	0.1	1.1350	1.1643	0	2	3	5	1.1658	1.0800	0	2	3	6
			0.3	1.1297	1.1902	0	2	3	5	1.1636	1.0903	0	2	3	6
			0.5	1.1170	1.2569	0	2	3	5	1.1583	1.1128	0	2	3	6
		1.1	0.1	1.1350	1.1643	0	1	2	3	1.1658	1.0800	0	2	3	6
			0.3	1.1297	1.1902	0	1	2	3	1.1636	1.0903	0	2	3	6
			0.5	1.1170	1.2569	0	2	3	5	1.1583	1.1128	0	2	3	6
		1.25	0.1	1.1350	1.1643	0	1	2	3	1.1658	1.0800	0	2	3	6
			0.3	1.1297	1.1902	0	2	3	5	1.1636	1.0903	0	2	3	6
			0.5	1.1170	1.2569	0	2	3	5	1.1583	1.1128	0	2	3	6
		1.5	0.1	1.1350	1.1643	0	1	2	3	1.1658	1.0800	0	2	3	6
			0.3	1.1297	1.1902	0	1	2	3	1.1636	1.0903	0	2	3	6
			0.5	1.1170	1.2569	0	1	2	3	1.1583	1.1128	0	2	3	6
	0.1	1.05	0.1	1.1280	1.1643	0	2	3	5	1.1572	1.0800	0	2	3	6
			0.3	1.1230	1.1902	0	2	3	5	1.1551	1.0903	0	2	3	6
			0.5	1.1110	1.2569	0	2	3	5	1.1501	1.1128	0	2	3	6
		1.1	0.1	1.1280	1.1643	0	1	2	3	1.1572	1.0800	0	2	3	6
			0.3	1.1230	1.1902	0	1	2	3	1.1551	1.0903	0	2	3	6
			0.5	1.1110	1.2569	0	2	3	5	1.1501	1.1128	0	2	3	6
		1.25	0.1	1.1280	1.1643	0	2	3	5	1.1572	1.0800	0	2	3	6
			0.3	1.1230	1.1902	0	2	3	5	1.1551	1.0903	0	2	3	6
			0.5	1.1110	1.2569	0	2	3	5	1.1501	1.1128	0	2	3	6
		1.5	0.1	1.1280	1.1643	0	1	2	3	1.1572	1.0800	0	2	3	6
			0.3	1.1230	1.1902	0	1	2	3	1.1551	1.0903	0	2	3	6
			0.5	1.1110	1.2569	0	1	2	3	1.1501	1.1128	0	2	3	6
4	0.05	1.05	0.1	1.2179	1.0692	0	1	2	4	1.2146	1.0468	0	2	5	8
			0.3	1.2134	1.0811	0	1	2	4	1.2113	1.0537	0	3	5	10
			0.5	1.2005	1.1120	0	1	2	4	1.2038	1.0695	0	1	2	4
		1.1	0.1	1.2179	1.0692	0	2	5	8	1.2146	1.0468	0	3	5	10
			0.3	1.2134	1.0811	0	2	5	8	1.2113	1.0537	0	2	5	8
			0.5	1.2005	1.1120	0	1	2	4	1.2038	1.0695	0	1	2	4
		1.25	0.1	1.2179	1.0692	0	2	5	8	1.2146	1.0468	0	1	2	4
			0.3	1.2134	1.0811	0	1	2	4	1.2113	1.0537	0	2	5	8
			0.5	1.2005	1.1120	0	1	2	4	1.2038	1.0695	0	2	5	8
		1.5	0.1	1.2179	1.0692	0	1	2	4	1.2539	1.0234	0	2	3	8
			0.3	1.2134	1.0811	0	2	5	8	1.2522	1.0270	0	2	3	8
			0.5	1.2005	1.1120	0	2	5	8	1.2038	1.0695	0	3	5	10
	0.1	1.05	0.1	1.2066	1.0692	0	2	5	8	1.2034	1.0468	0	2	5	8
			0.3	1.2023	1.0811	0	1	2	4	1.2004	1.0537	0	1	2	4
			0.5	1.1901	1.1120	0	3	5	10	1.1932	1.0695	0	1	2	4
		1.1	0.1	1.2066	1.0692	0	2	5	8	1.2034	1.0468	0	3	5	10
			0.3	1.2023	1.0811	0	2	5	8	1.2004	1.0537	0	2	5	8
			0.5	1.1901	1.1120	0	1	2	4	1.1932	1.0695	0	2	5	8
		1.25	0.1	1.2066	1.0692	0	2	5	8	1.2034	1.0468	0	1	2	4
			0.3	1.2023	1.0811	0	2	5	8	1.2004	1.0537	0	1	2	4
			0.5	1.1901	1.1120	0	2	5	8	1.1932	1.0695	0	3	5	10
		1.5	0.1	1.2066	1.0692	0	2	5	8	1.2034	1.0468	0	1	2	4
			0.3	1.2023	1.0811	0	2	5	8	1.2004	1.0537	0	2	5	8
			0.5	1.1901	1.1120	0	1	2	4	1.1932	1.0695	0	3	5	10

**Table 4:** Optimal parameters of the downward VSI RS MCV chart in minimizing ATS(*τ*) when $\text{AT}{\text{S}}_{\text{0}}$ = 500 and *v* = 2.

*G*	$d_{1}$	*τ*	$\gamma_{0}$	*n* = 5						*n* = 10					
				$d_{2}$	*K*	$S_{1}$	$S_{2}$	$S_{3}$	$S_{4}$	$d_{2}$	*K*	$S_{1}$	$S_{2}$	$S_{3}$	$S_{4}$
3	0.05	0.5	0.1	1.1593	0.6009	−1	−2	−2	−9	1.1532	0.8805	0	−2	−3	−6
			0.3	1.1593	0.5991	−1	−2	−2	−9	1.1532	0.8779	0	−2	−3	−6
			0.5	1.1595	0.5962	−1	−2	−2	−9	1.1578	0.8776	0	−1	−1	−3
		0.6	0.1	1.1593	0.6009	−1	−2	−2	−9	1.1570	0.8846	0	−1	−1	−3
			0.3	1.1593	0.5991	−1	−2	−2	−9	1.1578	0.8820	0	−1	−1	−3
			0.5	1.1595	0.5962	−1	−2	−2	−9	1.1578	0.8776	0	−1	−1	−3
		0.75	0.1	1.1593	0.6009	−1	−2	−2	−9	1.1605	0.7737	−1	−2	−3	−9
			0.3	1.1593	0.5991	−1	−2	−2	−9	1.1608	0.7700	−1	−2	−3	−9
			0.5	1.1595	0.5962	−1	−2	−2	−9	1.1611	0.7638	−1	−2	−3	−9
		0.9	0.1	1.1349	0.1311	−1	−2	−2	−8	1.1605	0.7737	−1	−2	−3	−9
			0.3	1.1349	0.1304	−1	−2	−2	−8	1.1608	0.7700	−1	−2	−3	−9
			0.5	1.1349	0.1294	−1	−2	−2	−8	1.1611	0.7638	−1	−2	−3	−9
	0.1	0.5	0.1	1.1510	0.6009	−1	−2	−2	−9	1.1454	0.8805	0	−2	−3	−6
			0.3	1.1511	0.5991	−1	−2	−2	−9	1.1452	0.8779	0	−2	−3	−6
			0.5	1.1512	0.5962	−1	−2	−2	−9	1.1460	0.8735	0	−2	−3	−6
		0.6	0.1	1.1510	0.6009	−1	−2	−2	−9	1.1488	0.8846	0	−1	−1	−3
			0.3	1.1511	0.5991	−1	−2	−2	−9	1.1496	0.8820	0	−1	−1	−3
			0.5	1.1512	0.5962	−1	−2	−2	−9	1.1496	0.8776	0	−1	−1	−3
		0.75	0.1	1.1510	0.6009	−1	−2	−2	−9	1.1522	0.7737	−1	−2	−3	−9
			0.3	1.1511	0.5991	−1	−2	−2	−9	1.1524	0.7700	−1	−2	−3	−9
			0.5	1.1512	0.5962	−1	−2	−2	−9	1.1528	0.7638	−1	−2	−3	−9
		0.9	0.1	1.1279	0.1311	−1	−2	−2	−8	1.1522	0.7737	−1	−2	−3	−9
			0.3	1.1279	0.1304	−1	−2	−2	−8	1.1524	0.7700	−1	−2	−3	−9
			0.5	1.1279	0.1294	−1	−2	−2	−8	1.1528	0.7638	−1	−2	−3	−9
4	0.05	0.5	0.1	1.2483	0.9286	0	−1	−1	−4	1.2424	0.9588	0	−2	−3	−8
			0.3	1.2483	0.9281	0	−1	−1	−4	1.2429	0.9579	0	−2	−3	−8
			0.5	1.2485	0.9271	0	−1	−1	−4	1.2435	0.9565	0	−2	−3	−8
		0.6	0.1	1.3163	0.1311	−1	−2	−2	−8	1.2424	0.9588	0	−2	−3	−8
			0.3	1.3163	0.1304	−1	−2	−2	−8	1.2518	0.9646	0	−1	−1	−4
			0.5	1.3164	0.1294	−1	−2	−2	−8	1.2519	0.9634	0	−1	−1	−4
		0.75	0.1	1.3163	0.1311	−1	−2	−2	−8	1.2513	0.9654	0	−1	−1	−4
			0.3	1.3163	0.1304	−1	−2	−2	−8	1.2518	0.9646	0	−1	−1	−4
			0.5	1.3164	0.1294	−1	−2	−2	−8	1.2519	0.9634	0	−1	−1	−4
		0.9	0.1	1.3163	0.1311	−1	−2	−2	−8	1.3164	0.4127	−1	−2	−2	−8
			0.3	1.3163	0.1304	−1	−2	−2	−8	1.3164	0.4079	−1	−2	−2	−8
			0.5	1.3164	0.1294	−1	−2	−2	−8	1.3164	0.4000	−1	−2	−2	−8
	0.1	0.5	0.1	1.2354	0.9286	0	−1	−1	−4	1.1850	0.9199	0	−1	−2	−4
			0.3	1.2353	0.9281	0	−1	−1	−4	1.2302	0.9579	0	−2	−3	−8
			0.5	1.2355	0.9271	0	−1	−1	−4	1.2308	0.9565	0	−2	−3	−8
		0.6	0.1	1.2998	0.1311	−1	−2	−2	−8	1.2298	0.9588	0	−2	−3	−8
			0.3	1.2998	0.1304	−1	−2	−2	−8	1.2302	0.9579	0	−2	−3	−8
			0.5	1.2998	0.1294	−1	−2	−2	−8	1.2308	0.9565	0	−2	−3	−8
		0.75	0.1	1.2998	0.1311	−1	−2	−2	−8	1.2383	0.9654	0	−1	−1	−4
			0.3	1.2998	0.1304	−1	−2	−2	−8	1.2386	0.9646	0	−1	−1	−4
			0.5	1.2998	0.1294	−1	−2	−2	−8	1.2388	0.9634	0	−1	−1	−4
		0.9	0.1	1.2998	0.1311	−1	−2	−2	−8	1.2999	0.4127	−1	−2	−2	−8
			0.3	1.2998	0.1304	−1	−2	−2	−8	1.2999	0.4079	−1	−2	−2	−8
			0.5	1.2998	0.1294	−1	−2	−2	−8	1.2999	0.4000	−1	−2	−2	−8

downward charts, respectively, when $\text{AT}{\text{S}}_{\text{0}}=\text{ 500}$. Note that *τ* ∈ {1.05, 1.1, 1.25, 1.5} and {0.5, 0.6, 0.75, 0.9} are adopted for the upward and downward charts, respectively. In Tables 1–4, *v* = 2, *n* ∈ {5, 10}, $d_{\text{1}}$ ∈ {0.05, 0.1}, *G* ∈ {3, 4}, $\gamma_{\text{0}}$ ∈ {0.1, 0.3, 0.5} are considered. For example, when the upward VSI RS MCV chart is optimally designed to minimize ATS(1.1), i.e., for *τ* = 1.1, when $\text{AT}{\text{S}}_{\text{0}}=\text{ 200}$,v = 2, n=5, G=3, $d_{\text{1}}=\text{0}.\text{0}\text{5}$and $\gamma_{\text{0}}=\text{0}.\text{3}$, the chart’s optimal parameter and score combination is $\left(d_{\text{2}},K,\left\{S_{\text{1}},S_{\text{2}},S_{\text{3}},S_{\text{4}}\right\}\right)$ = (1.1878, 1.1068, {0, 2, 3, 5}) (see Table 1). Additionally, Table 5 presents the optimal parameter and score combination that minimizes EATS$\left(\tau_{\text{min}},\tau_{\text{max}}\right)$, based on the above-mentioned *v*, *n*, $d_{\text{1}}$, *G*, $\gamma_{\text{0}}$ and $\text{AT}{\text{S}}_{\text{0}}$ values. The accuracy of the entries in Tables 1–5 has been verified with simulation conducted using the Statistical Analysis System (SAS) software.

**Table 5:** Optimal parameters of the VSI RS MCV chart in minimizing $\text{EATS}\left(\tau_{\text{min}},\tau_{\text{max}}\right)$ when>.v=2

*n*	*G*	$d_{1}$	$\gamma_{0}$	Upward chart												Downward chart											
				${𝐀𝐓𝐒}_{0}$ = 200						${𝐀𝐓𝐒}_{0}$ = 500						${𝐀𝐓𝐒}_{0}$ = 200						${𝐀𝐓𝐒}_{0}$ = 500					
				$\left(\tau_{𝐦𝐢𝐧},\tau_{𝐦𝐚𝐱}\right)$ = (1, 2)						$\left(\tau_{𝐦𝐢𝐧},\tau_{𝐦𝐚𝐱}\right)$ = (1, 2)						$\left(\tau_{𝐦𝐢𝐧},\tau_{𝐦𝐚𝐱}\right)$ = (0.5, 1)						$\left(\tau_{𝐦𝐢𝐧},\tau_{𝐦𝐚𝐱}\right)$ = (0.5, 1)					
				$d_{2}$	*K*	$S_{1}$	$S_{2}$	$S_{3}$	$S_{4}$	$d_{2}$	*K*	$S_{1}$	$S_{2}$	$S_{3}$	$S_{4}$	$d_{2}$	*K*	$S_{1}$	$S_{2}$	$S_{3}$	$S_{4}$	$d_{2}$	*K*	$S_{1}$	$S_{2}$	$S_{3}$	$S_{4}$
5	3	0.05	0.1	1.1915	1.0935	0	1	2	3	1.1350	1.1643	0	1	2	3	1.1778	0.7489	−1	−2	−2	−8	1.1349	0.1311	−1	−2	−2	−8
			0.3	1.1878	1.1068	0	2	3	5	1.1297	1.1902	0	1	2	3	1.1778	0.7472	−1	−2	−2	−8	1.1349	0.1304	−1	−2	−2	−8
			0.5	1.1796	1.1384	0	1	2	3	1.1170	1.2569	0	1	2	3	1.1778	0.7443	−1	−2	−2	−8	1.1349	0.1294	−1	−2	−2	−8
		0.10	0.1	1.1817	1.0935	0	1	2	3	1.1280	1.1643	0	2	3	5	1.1688	0.7489	−1	−2	−2	−8	1.1279	0.1311	−1	−2	−2	−8
			0.3	1.1782	1.1068	0	2	3	5	1.1230	1.1902	0	1	2	3	1.1688	0.7472	−1	−2	−2	−8	1.1279	0.1304	−1	−2	−2	−8
			0.5	1.1705	1.1384	0	2	5	6	1.1110	1.2569	0	2	3	5	1.1688	0.7443	−1	−2	−2	−8	1.1279	0.1294	−1	−2	−2	−8
	4	0.05	0.1	1.2924	1.0046	0	2	5	8	1.2179	1.0692	0	3	5	10	1.381	0.7489	−1	−2	−2	−8	1.3163	0.1311	−1	−2	−2	−8
			0.3	1.2916	1.0054	0	2	5	8	1.2134	1.0811	0	3	5	10	1.381	0.7472	−1	−2	−2	−8	1.3163	0.1304	−1	−2	−2	−8
			0.5	1.2911	1.0070	0	2	5	8	1.2005	1.1120	0	1	2	4	1.3811	0.7443	−1	−2	−2	−8	1.3164	0.1294	−1	−2	−2	−8
		0.10	0.1	1.2774	1.0046	0	2	5	8	1.2066	1.0692	0	1	2	4	1.3612	0.7489	−1	−2	−2	−8	1.2998	0.1311	−1	−2	−2	−8
			0.3	1.2765	1.0054	0	1	2	4	1.2023	1.0811	0	3	5	10	1.3613	0.7472	−1	−2	−2	−8	1.2998	0.1304	−1	−2	−2	−8
			0.5	1.2761	1.0070	0	1	2	4	1.1901	1.1120	0	1	2	4	1.3613	0.7443	−1	−2	−2	−8	1.2998	0.1294	−1	−2	−2	−8
10	3	0.05	0.1	1.1790	1.0619	0	1	2	3	1.1572	1.0800	0	2	3	6	1.2115	0.9400	−1	−2	−3	−9	1.1605	0.7737	−1	−2	−3	−9
			0.3	1.2230	1.0368	0	2	3	6	1.1551	1.0903	0	2	3	6	1.2117	0.9387	−1	−2	−3	−9	1.1608	0.7700	−1	−2	−3	−9
			0.5	1.2204	1.0451	0	2	3	6	1.1501	1.1128	0	2	3	6	1.2119	0.9366	−1	−2	−3	−9	1.1611	0.7638	−1	−2	−3	−9
		0.10	0.1	1.2362	1.0328	0	2	3	6	1.1658	1.0800	0	2	3	6	1.2008	0.9400	−1	−2	−3	−9	1.1522	0.7737	−1	−2	−3	−9
			0.3	1.2350	1.0368	0	2	3	6	1.1636	1.0903	0	2	3	6	1.2008	0.9387	−1	−2	−3	−9	1.1524	0.7700	−1	−2	−3	−9
			0.5	1.2918	1.0045	0	2	5	8	1.1583	1.1128	0	2	3	6	1.2011	0.9366	−1	−2	−3	−9	1.1528	0.7638	−1	−2	−3	−9
	4	0.05	0.1	1.2771	1.0031	0	1	2	4	1.2146	1.0468	0	3	5	10	1.383	0.8702	−1	−2	−2	−8	1.3164	0.4127	−1	−2	−2	−8
			0.3	1.2769	1.0036	0	3	5	10	1.2113	1.0537	0	3	5	10	1.383	0.8673	−1	−2	−2	−8	1.3164	0.4079	−1	−2	−2	−8
			0.5	1.2768	1.0045	0	2	5	8	1.2038	1.0695	0	3	5	10	1.3746	0.8420	−1	−2	−3	−8	1.3164	0.4000	−1	−2	−3	−8
		0.10	0.1	1.2921	1.0031	0	2	5	8	1.2034	1.0468	0	3	5	10	1.3548	0.8500	−1	−2	−3	−8	1.2999	0.4127	−1	−2	−3	−8
			0.3	1.2919	1.0036	0	3	5	10	1.2004	1.0537	0	3	5	10	1.3549	0.8470	−1	−2	−3	−8	1.2999	0.4079	−1	−2	−3	−8
			0.5	1.2918	1.0045	0	2	5	8	1.1932	1.0695	0	3	5	10	1.3553	0.8420	−1	−2	−3	−8	1.2999	0.4000	−1	−2	−3	−8

## 5. Performance evaluation and comparison

An evaluation of the VSI RS MCV chart is made by comparing its performance with that of the existing MCV and RS MCV charts, in terms of the steady state ATS(*τ*), SDTS(*τ*) and EATS$\left(\tau_{\text{min}},\tau_{\text{max}}\right)$ performance criteria, where *τ* ≠ 1. Tables 6–10 provide the comparative results for different $\text{AT}{\text{S}}_{\text{0}}$, *n*, *G*, $d_{\text{1}}$, *τ* and $\gamma_{\text{0}}$ combinations when *v* = 2. The ATS(τ) and SDTS(τ) values of all the charts decrease as the shift size τincreases (i) from 1.05 to 1.5 for the upward charts (see Tables 6 and 9) or (ii) from 0.9 to 0.5 for the downward charts (see Tables 7 and 10) but the VSI RS MCV chart always has the smallest ATS(τ) and SDTS(τ) values among all the charts for the same shift size *τ*. This outcome indicates that the proposed VSI RS MCV chart beats the existing MCV and RS MCV charts in detecting shifts based on the ATS(τ) and SDTS(τ) criteria. For instance, in Table 6, when $\text{AT}{\text{S}}_{\text{0}}$ = 200, *τ* = 1.1, $\gamma_{\text{0}}$= 0.1, *n* = 5 and *G* = 3, ATS(1.1) are 50.42 and 50.73 when $d_{\text{1}}$ are 0.05 and 0.1, respectively, for the proposed upward VSI RS MCV chart. In contrast, the existing upward MCV and RS MCV

**Table 6:** ATS(*τ*) values for the upward MCV, RS MCV and VSI RS MCV charts for different *n*, $\begin{array}{c} AT{𝐒}_{0} \end{array}$, $\begin{array}{c} \gamma_{0} \end{array}$ and *G* values.

$𝐀𝐓{𝐒}_{0}$	τ	$\gamma_{0}$	n=5						n=10					
			MCV	RS MCV	VSI RS MCV				MCV	RS MCV	VSI RS MCV			
					ATS(*τ*)						ATS(*τ*)			
			ATS(*τ*)	ATS(*τ*)	*G* = 3		*G* = 4		ATS(*τ*)	ATS(*τ*)	*G* = 3		*G* = 4	
					$d_{1}$ = 0.05	$d_{1}$ = 0.1	$d_{1}$ = 0.05	$d_{1}$ = 0.1			$d_{1}$ = 0.05	$d_{1}$ = 0.1	$d_{1}$ = 0.05	$d_{1}$ = 0.1
200	1.05	0.1	114.26	99.04	94.71	94.98	92.76	93.10	93.30	71.53	66.39	66.72	63.99	64.39
		0.3	117.50	102.39	98.13	98.40	96.20	96.53	97.75	75.83	70.84	71.21	68.39	68.78
		0.5	124.81	109.35	105.38	105.63	103.36	103.68	106.23	83.90	78.93	79.29	76.56	76.94
	1.1	0.1	70.70	55.60	50.42	50.73	48.67	49.04	49.12	32.28	27.15	27.46	25.56	25.91
		0.3	74.49	58.96	53.77	54.08	51.97	52.34	53.47	35.57	30.42	30.73	28.65	29.02
		0.5	83.32	66.23	61.13	61.44	59.13	59.51	62.19	42.09	36.71	37.08	34.81	35.19
	1.25	0.1	23.26	16.55	12.63	12.85	11.91	12.16	11.68	7.29	4.43	4.58	4.03	4.20
		0.3	25.92	18.39	14.27	14.50	13.49	13.75	13.81	8.46	5.36	5.53	4.89	5.08
		0.5	32.65	22.70	18.19	18.44	17.20	17.49	18.55	10.97	7.39	7.61	6.78	7.00
	1.5	0.1	7.03	5.35	3.26	3.38	3.01	3.14	2.63	2.09	0.85	0.93	0.74	0.82
		0.3	8.37	6.22	3.90	4.02	3.61	3.76	3.42	2.58	1.11	1.20	0.98	1.08
		0.5	12.01	8.36	5.49	5.64	5.11	5.29	5.31	3.61	1.71	1.82	1.54	1.65
500	1.05	0.1	262.41	216.19	209.21	209.75	203.33	204.01	210.73	148.89	140.04	140.74	134.35	135.12
		0.3	271.24	225.50	218.44	218.95	212.93	213.59	222.32	160.27	151.08	151.78	145.79	146.56
		0.5	292.37	246.66	239.74	240.21	234.55	235.19	245.14	182.61	172.41	173.09	168.13	168.88
	1.1	0.1	150.63	108.44	100.24	100.80	95.45	96.14	101.57	57.87	49.85	50.43	46.31	46.92
		0.3	160.18	116.68	108.34	108.90	103.59	104.28	111.97	65.28	56.79	57.39	53.21	53.84
		0.5	184.09	136.31	127.94	128.49	122.85	123.57	133.70	80.87	71.25	71.88	67.79	68.48
	1.25	0.1	41.89	25.82	20.07	20.41	18.55	18.94	20.01	10.22	6.30	6.55	5.63	5.87
		0.3	47.55	29.26	23.19	23.54	21.52	21.93	24.21	12.13	7.80	8.07	7.02	7.29
		0.5	63.17	38.14	31.47	31.86	29.20	29.67	34.09	16.52	11.29	11.61	10.31	10.64
	1.5	0.1	10.79	7.19	4.35	4.50	3.95	4.12	3.86	2.68	1.07	1.17	0.93	1.03
		0.3	13.17	8.50	5.31	5.48	4.84	5.03	5.15	3.32	1.42	1.53	1.25	1.36
		0.5	20.38	11.93	7.99	8.20	7.27	7.51	8.46	4.75	2.25	2.39	2.01	2.16

**Table 7:** ATS(*τ*) values for the downward MCV, RS MCV and VSI RS MCV charts for different *n*, $\begin{array}{c} AT{𝐒}_{0} \end{array}$, $\begin{array}{c} \gamma_{0} \end{array}$ and *G* values.

$𝐀𝐓{𝐒}_{0}$	*τ*	$\gamma_{0}$	*n* = 5						*n *= 10					
			MCV	RS MCV	VSI RS MCV				MCV	RS MCV	VSI RS MCV			
			ATS(*τ*)	ATS(*τ*)	ATS(*τ*)				ATS(*τ*)	ATS(*τ*)	ATS(*τ*)			
					*G* = 3		*G* = 4				*G* = 3		*G* = 4	
					$d_{1}$ = 0.05	$d_{1}$ = 0.1	$d_{1}$ = 0.05	$d_{1}$ = 0.1			$d_{1}$ = 0.05	$\;d_{1}$ = 0.1	$d_{1}$ = 0.05	$d_{1}$ = 0.1
200	0.5	0.1	25.82	4.98	1.10	1.26	0.72	0.89	2.57	2.28	0.14	0.21	0.11	0.18
		0.3	27.16	5.15	1.20	1.36	0.79	0.97	2.93	2.36	0.16	0.23	0.12	0.19
		0.5	29.73	5.49	1.41	1.58	0.95	1.14	3.65	2.53	0.20	0.28	0.15	0.24
	0.6	0.1	44.12	7.81	2.82	3.03	2.23	2.48	7.46	3.35	0.45	0.56	0.32	0.44
		0.3	46.06	8.16	3.06	3.28	2.48	2.74	8.35	3.54	0.51	0.63	0.37	0.49
		0.5	49.71	8.89	3.60	3.83	3.01	3.27	10.08	3.86	0.65	0.78	0.48	0.61
	0.75	0.1	85.12	20.09	13.03	13.36	11.68	12.08	29.32	7.91	3.10	3.31	2.53	2.77
		0.3	87.65	21.31	14.15	14.49	12.72	13.13	31.80	8.59	3.58	3.80	2.95	3.20
		0.5	92.24	23.81	16.46	16.82	14.89	15.31	36.32	9.90	4.54	4.77	3.86	4.13
	0.9	0.1	145.78	74.10	67.34	67.67	64.32	64.81	96.83	39.40	31.36	31.75	29.49	29.96
		0.3	147.63	76.94	70.26	70.58	67.24	67.72	100.47	42.31	34.25	34.64	32.27	32.75
		0.5	150.86	82.31	75.79	76.10	72.79	73.26	106.50	47.70	39.64	40.04	37.49	37.98
500	0.5	0.1	64.12	6.66	1.82	2.05	1.20	1.44	5.48	2.68	0.22	0.31	0.16	0.26
		0.3	67.46	6.92	1.92	2.16	1.32	1.56	6.24	2.81	0.25	0.34	0.18	0.28
		0.5	73.88	7.49	2.16	2.40	1.58	1.84	7.80	3.06	0.32	0.42	0.22	0.34
	0.6	0.1	109.86	10.66	4.09	4.39	3.46	3.81	16.36	4.17	0.70	0.83	0.49	0.64
		0.3	114.71	11.21	4.46	4.77	3.80	4.16	18.39	4.41	0.79	0.94	0.56	0.72
		0.5	123.86	12.38	5.28	5.61	4.55	4.93	22.37	4.87	1.00	1.16	0.72	0.90
	0.75	0.1	212.67	31.70	21.31	21.83	19.03	19.65	68.34	11.13	4.77	5.07	4.01	4.35
		0.3	219.01	33.95	23.35	23.88	20.90	21.54	74.40	12.07	5.45	5.76	4.73	5.10
		0.5	230.54	38.64	27.67	28.23	24.87	25.54	85.44	14.05	6.92	7.25	6.34	6.75
	0.9	0.1	365.11	149.64	136.12	136.79	130.06	131.05	237.02	72.12	58.47	59.14	56.79	57.66
		0.3	369.76	156.61	143.15	143.82	137.04	138.04	246.24	78.50	64.58	65.26	62.82	63.72
		0.5	377.87	169.99	156.67	157.34	150.53	151.51	261.51	90.55	76.23	76.94	74.41	75.34

**Table 8:** $\text{EATS}\left(\tau_{\text{min}},\tau_{\text{max}}\right)$ values of the upward and downward MCV, RS MCV and VSI RS MCV charts for different *n*, $\text{AT}{\text{S}}_{\text{0}}$, $\gamma_{\text{0}}$ and *G* values.

*n*	$𝐀𝐓{𝐒}_{0}$	$\gamma_{0}$	Upward chart						Downward chart					
			$\left(\tau_{𝐦𝐢𝐧},\tau_{𝐦𝐚𝐱}\right)$ = (1, 2)						$\left(\tau_{𝐦𝐢𝐧},\tau_{𝐦𝐚𝐱}\right)$ = (0.5, 1)					
			MCV	RS MCV	VSI RS MCV				MCV	RS MCV	VSI RS MCV			
			𝐄𝐀𝐓𝐒 $\left(\tau_{𝐦𝐢𝐧},\tau_{𝐦𝐚𝐱}\right)$	𝐄𝐀𝐓𝐒 $\left(\tau_{𝐦𝐢𝐧},\tau_{𝐦𝐚𝐱}\right)$	$𝐄𝐀𝐓𝐒\left(\tau_{𝐦𝐢𝐧},\tau_{𝐦𝐚𝐱}\right)$				𝐄𝐀𝐓𝐒 $\left(\tau_{𝐦𝐢𝐧},\tau_{𝐦𝐚𝐱}\right)$	𝐄𝐀𝐓𝐒 $\left(\tau_{𝐦𝐢𝐧},\tau_{𝐦𝐚𝐱}\right)$	$𝐄𝐀𝐓𝐒\left(\tau_{𝐦𝐢𝐧},\tau_{𝐦𝐚𝐱}\right)$			
					*G* = 3		*G* = 4				*G* = 3		*G* = 4	
					$d_{1}$ = 0.05	$d_{1}$ = 0.1	$d_{1}$ = 0.05	$d_{1}$ = 0.1			$d_{1}$ = 0.05	$d_{1}$ = 0.1	$d_{1}$ = 0.05	$d_{1}$ = 0.1
5	200	0.1	23.31	19.29	16.77	16.91	16.25	16.41	94.22	41.66	36.89	37.16	35.31	35.66
		0.3	25.00	20.56	17.87	18.02	17.32	17.49	96.15	42.96	38.17	38.44	36.56	36.92
		0.5	29.30	23.51	20.43	20.60	19.77	19.97	99.66	45.49	40.65	40.92	39.00	39.36
	500	0.1	48.51	37.87	34.25	34.47	33.03	33.29	235.64	86.22	78.07	78.51	75.06	75.66
		0.3	52.05	40.39	36.51	36.74	35.25	35.52	240.48	89.15	80.92	81.36	77.84	78.45
		0.5	61.79	46.66	42.21	42.47	40.72	41.03	249.29	94.89	86.50	86.95	83.31	83.93
10	200	0.1	16.04	12.39	10.52	10.63	10.12	10.24	51.46	26.12	22.08	22.30	21.18	21.44
		0.3	17.44	13.39	11.34	11.48	10.91	11.04	53.53	27.30	23.19	23.41	22.25	22.53
		0.5	20.46	15.42	12.52	13.16	12.52	12.67	57.16	29.48	25.24	25.48	24.25	24.53
	500	0.1	34.13	24.58	21.87	22.06	21.06	21.24	125.09	53.86	47.16	47.52	46.26	46.75
		0.3	37.05	26.57	23.56	23.76	22.74	22.94	130.19	56.43	49.58	49.95	48.68	49.17
		0.5	43.61	30.75	27.09	27.31	26.26	26.50	139.13	61.18	54.08	54.46	53.20	53.71

**Table 9:** SDTS(*τ*) values of the upward MCV, RS MCV and VSI RS MCV charts for different *n*, $\text{AT}{\text{S}}_{\text{0}}$, $\gamma_{\text{0}}$ and *G* values.

$𝐀𝐓{𝐒}_{0}$	*τ*	$\gamma_{0}$	*n* = 5						*n* = 10					
			MCV	RS MCV	VSI RS MCV				MCV	RS MCV	VSI RS MCV			
			SDTS(*τ*)	SDTS(*τ*)	SDTS(*τ*)				SDTS(*τ*)	SDTS(*τ*)	SDTS(*τ*)			
					*G* = 3		*G* = 4				*G* = 3		*G* = 4	
					$d_{1}$ = 0.05	$d_{1}$ = 0.1	$d_{1}$ = 0.05	$d_{1}$ = 0.1			$d_{1}$ = 0.05	$d_{1}$ = 0.1	*d*_1_ = 0.05	*d*_1_ = 0.1
200	1.05	0.1	114.75	98.94	96.59	96.75	94.80	95.00	93.80	71.18	68.03	68.25	65.78	66.05
		0.3	118.00	102.31	100.01	100.17	98.26	98.46	98.25	75.51	72.76	72.99	70.22	70.48
		0.5	125.31	109.31	107.23	107.38	105.47	105.66	106.73	83.63	80.91	81.14	78.46	78.72
	1.1	0.1	71.20	55.22	51.91	52.12	50.27	50.51	49.62	31.62	28.36	28.55	26.86	27.08
		0.3	74.98	58.60	55.27	55.47	53.61	53.85	53.97	34.93	31.67	31.86	30.00	30.24
		0.5	83.82	65.90	62.66	62.86	60.84	61.09	62.69	41.50	38.18	38.42	36.25	36.50
	1.25	0.1	23.75	15.99	13.58	13.70	12.93	13.07	12.17	6.67	5.15	5.21	4.80	4.87
		0.3	26.41	17.83	15.25	15.38	14.54	14.69	14.30	7.80	6.15	6.19	5.69	5.78
		0.5	33.14	22.10	19.22	19.37	18.34	18.51	19.04	10	8.24	8.35	7.65	7.76
	1.5	0.1	7.52	5.09	3.96	4.00	3.76	3.81	3.09	2.04	1.46	1.46	1.41	1.41
		0.3	8.86	5.90	4.61	4.66	4.39	4.44	3.89	2.44	1.72	1.74	1.65	1.66
		0.5	12.50	7.92	6.24	6.31	5.93	6.01	5.78	3.23	2.34	2.37	2.21	2.23
500	1.05	0.1	262.91	215.88	211.14	211.56	205.42	205.97	211.23	148.26	141.95	142.49	136.15	136.77
		0.3	271.74	225.24	220.33	220.72	215.00	215.53	222.82	159.70	153.01	153.55	147.61	148.24
		0.5	292.87	246.51	241.52	241.88	236.53	237.05	245.64	182.15	174.36	174.89	169.97	170.59
	1.1	0.1	151.13	107.74	101.72	102.16	97.04	97.59	102.07	56.82	51.15	51.58	47.57	48.03
		0.3	160.68	116.04	109.81	110.25	105.19	105.75	112.47	64.28	58.14	58.59	54.50	55.00
		0.5	184.58	135.79	129.38	129.82	124.45	125.04	134.20	79.96	72.66	73.15	69.16	69.71
	1.25	0.1	42.38	24.89	20.96	21.18	19.50	19.76	20.51	9.23	6.97	7.09	6.32	6.44
		0.3	48.04	28.34	24.10	24.34	22.50	22.78	24.70	11.10	8.50	8.64	7.74	7.88
		0.5	63.66	37.24	32.41	32.69	30.23	30.57	34.59	15.41	12.05	12.24	11.09	11.29
	1.5	0.1	11.27	6.68	4.97	5.04	4.62	4.70	4.33	2.38	1.61	1.63	1.51	1.53
		0.3	13.66	7.94	5.95	6.03	5.53	5.62	5.62	2.91	1.95	1.98	1.83	1.85
		0.5	20.87	11.14	8.66	8.77	7.99	8.13	8.94	4.07	2.79	2.84	2.59	2.63

charts yield values of 70.70 and 55.60, respectively, indicating that the proposed upward VSI RS MCV chart exhibits a lower ATS(1.1) value than the existing upward charts.

A careful scrutiny of Tables 6 and 7 reveals that the outperformance of the proposed VSI RS MCV chart over the existing MCV and RS MCV charts increases as the shift size *τ* becomes larger. For illustration, consider $\text{AT}{\text{S}}_{\text{0}}=\text{200}$, n=5, $\gamma_{\text{0}}=\text{0}.\text{3}$, G=3 and $d_{\text{1}}=\text{0}.\text{0}\text{5}$. When τ=1.05 (small shift), ATS(1.05) of the VSI RS MCV, MCV and RS MCV charts are 98.13, 117.50 and 102.39 (see Table 6), respectively, i.e., the VSI RS MCV chart is 19.74% and 4.34% faster than the MCV and RS MCV charts in detecting the shift *τ* = 1.05. By keeping the aforementioned parameter values, when *τ* = 1.5 (large shift), ATS(1.5) = 3.90, 8.37 and 6.22 (see Table 6) for the VSI RS MCV, MCV and RS MCV charts, respectively, where the VSI RS MCV chart is 114.62% and 59.49% quicker than the MCV and RS MCV charts in the detection of the shift *τ* = 1.5. This example clearly illustrates that the proposed VSI RS MCV chart demonstrates quicker process shift detection speed versus its existing counterparts as *τ* increases.

The EATS$\left(\tau_{\text{min}},\tau_{\text{max}}\right)$ results in Table 8 also provide similar trend to that of the ATS(*τ*) results in Tables 6 and 7, where the VSI RS MCV chart surpasses its existing MCV and RS MCV counterparts, in detecting shifts for all shift intervals $\left(\tau_{\text{min}},\tau_{\text{max}}\right)$, irrespective of the $\text{AT}{\text{S}}_{\text{0}}$, *n*, *G*, $d_{\text{1}}$ and $\gamma_{\text{0}}$ combination. For example, when $\left(\tau_{\text{min}},\tau_{\text{max}}\right)$ = (1, 2), $\text{AT}{\text{S}}_{\text{0}}$ = 200, *n* = 5, *G* = 4, $d_{\text{1}}$ = 0.1 and $\gamma_{\text{0}}$ = 0.5, EATS(1, 2) = 19.97, 29.30 and 23.51 (see Table 8), for the VSI RS MCV, MCV and RS MCV charts, respectively, where the VSI RS MCV chart has the smallest EATS(1, 2) value, indicating that it gives the quickest response to shifts in the interval $\left(\tau_{\text{min}},\tau_{\text{max}}\right)$ = (1, 2).

The SDTS criterion measures the variability in the time to signal distribution. Thus, a chart with a smaller SDTS is desirable as it signifies lesser variability in the time to signal distribution of the chart, resulting in a better performing chart. Similar to the ATS(*τ*) results discussed earlier, the three considered upward and downward charts also show similar trends, where the VSI RS MCV charts have the smallest SDTS(*τ*) value among all the charts under comparison, for any shift size *τ* (see Tables 9 and 10). As an example, consider the downward charts in Table 10 when $\text{AT}{\text{S}}_{\text{0}}$ = 200, *τ* = 0.6, $\gamma_{\text{0}}$ = 0.5, *n* = 5, *G* = 4 and $d_{\text{1}}$ = 0.1. Here, SDTS(0.6) = 3.67, 50.21 and 5.66 for the VSI RS MCV, MCV and RS MCV charts, respectively, where the VSI RS MCV chart has the smallest SDTS(0.6) value, indicating that its time to signal distribution has lower variability compared to that of the MCV and RS MCV charts, hence, the VSI RS MCV chart gives the most desirable SDTS performance.

The larger the shift size, the more pronounced the superiority of the VSI RS MCV chart’s SDTS performance becomes. To demonstrate, consider $\text{AT}{\text{S}}_{\text{0}}$ = 200, *n* = 5, $\gamma_{\text{0}}$ = 0.3, *G* = 3 and $d_{\text{1}}$ = 0.05 in Table 9. With these parameters, when *τ* = 1.05 (small shift), SDTS (1.05) = 100.01, 118.00 and 102.31, for the VSI RS MCV, MCV and RS MCV charts, respectively, where the SDTS (1.05) value of the VSI RS MCV chart is 15.25% and 2.25% lower than that of the MCV and RS MCV charts. Similarly, when *τ* = 1.5 (large shift), the SDTS(1.5) values are 4.61, 8.86 and 5.90 for the VSI RS MCV, MCV and RS MCV charts, respectively, indicating that the SDTS(1.5) value of the VSI RS MCV chart is 47.97% and 21.86% lower than those of the MCV and RS MCV charts. This example clearly shows that the reduction in the SDTS value of the proposed VSI RS MCV chart, in comparison to the existing MCV and RS MCV charts, increases with the shift size *τ*.

It is found that the ATS(*τ*), SDTS(*τ*) and EATS$\left(\tau_{\text{min}},\tau_{\text{max}}\right)$ values of all the charts increase with $\gamma_{\text{0}}$, indicating that the charts’ performances deteriorate as $\gamma_{\text{0}}$ increases. Besides that, it is seen that the proposed VSI RS MCV charts perform slightly better in terms of the ATS(*τ*) and EATS$\left(\tau_{\text{min}},\tau_{\text{max}}\right)$ criteria when $d_{\text{1}}=$ 0.05, instead of $d_{\text{1}}=\text{0}.\text{1}$. For example, in Table 7 when $\text{AT}{\text{S}}_{\text{0}}$ = 500, *τ* = 0.6, $\gamma_{\text{0}}$ = 0.1, *n* = 5 and *G* = 3, ATS(0.6) = 4.09 and 4.39 for the downward VSI RS MCV chart when $d_{\text{1}}$ = 0.05 and 0.1, respectively, where the chart with $d_{\text{1}}$ = 0.05 has a slightly lower ATS(0.6) value. Moreover, it is noticed that the proposed VSI RS MCV charts have lower ATS(*τ*), SDTS(*τ*) and $\text{EATS}\left(\tau_{\text{min}},\tau_{\text{max}}\right)$ values as the switching threshold *G* increases, for the same ($\text{AT}{\text{S}}_{\text{0}}$, *τ*,$\gamma_{\text{0}}$, *n*) combination, which means that a larger value of *G* enhances the charts’ performances. Therefore, the proposed VSI RS MCV charts generally become more efficient by using the combination $d_{\text{1}}$= 0.05 and *G* = 4. In addition, it is worthy to note that increasing the sample size from *n* = 5–10 results in better performance for all the charts, as the ATS(*τ*), SDTS(*τ*) and EATS$\left(\tau_{\text{min}},\tau_{\text{max}}\right)$ values decrease with an increase in *n*. Hence, the speed of a chart in responding to process shifts becomes quicker by employing a larger *n*.

Based on the findings in this section, it can be concluded that the proposed VSI RS MCV chart prevails over the existing MCV and RS MCV charts, in terms of the ATS(*τ*), SDTS(*τ*) and EATS$\left(\tau_{\text{min}},\tau_{\text{max}}\right)$ performance criteria.

## 6. An illustrative example

A dataset involving the inside diameter measurements of steel sleeve adopted from [13] is used to demonstrate the construction of the proposed 4 regions upward and downward VSI RS MCV charts. In this dataset, two quality characteristics, i.e., diameters A and B, whose observations are represented as $X_{\text{1}}$ and $X_{\text{2}}$, respectively, are monitored concurrently. Table 11 shows the Phase-I dataset with 20 samples, each with size *n* = 5, and the computed sample MCVs, ${\hat{\gamma }}_{t}$ (*t* = 1, 2, …, 20) for each sample. From the 20 ${\hat{\gamma }}_{t}$ values of the Phase-I process in Table 11, the in-control MCV is estimated as ${\hat{\gamma }}_{\text{0}}=\sqrt{(\text{1}/\text{20}sum_{t=\text{1}}^{\text{20}}\;{\hat{\gamma }}_{t}^{\text{2}}}=\text{0}.\text{089115}$.

**Table 10:** SDTS(*τ*) values of the downward MCV, RS MCV and VSI RS MCV charts for different *n*, $\text{AT}{\text{S}}_{\text{0}}$, $\gamma_{\text{0}}$ and *G* values.

$𝐀𝐓{𝐒}_{0}$	τ	$\gamma_{0}$	*n* = 5						*n* = 10					
			MCV	RS MCV	VSI RS MCV				MCV	RS MCV	VSI RS MCV			
			SDTS(*τ*)	SDTS(*τ*)	SDTS(*τ*)				SDTS(*τ*)	SDTS(*τ*)	SDTS(*τ*)			
					*G* = 3		*G* = 4				*G* = 3		*G* = 4	
					$d_{1}$ = 0.05	$d_{1}$ = 0.1	$d_{1}$ = 0.05	$d_{1}$ = 0.1			$d_{1}$ = 0.05	$d_{1}$ = 0.1	$d_{1}$ = 0.05	$d_{1}$ = 0.1
200	0.5	0.1	26.31	2.16	1.56	1.59	1.20	1.23	3.03	0.83	0.68	0.68	0.68	0.68
		0.3	27.65	2.31	1.66	1.69	1.28	1.31	3.39	0.88	0.69	0.69	0.69	0.69
		0.5	30.23	2.63	1.88	1.91	1.45	1.49	4.12	1.00	0.73	0.73	0.71	0.72
	0.6	0.1	44.62	4.55	3.10	3.17	2.83	2.91	7.95	1.58	0.95	0.97	0.85	0.86
		0.3	46.55	4.91	3.36	3.43	3.10	3.18	8.83	1.51	1.01	1.02	0.89	0.91
		0.5	50.21	5.66	3.92	4.01	3.56	3.67	10.57	1.75	1.15	1.16	1.00	1.02
	0.75	0.1	85.62	17.07	13.77	13.95	12.70	12.92	29.81	5.39	3.71	3.78	3.17	3.25
		0.3	88.14	18.31	14.93	15.11	13.79	14.01	32.30	6.06	4.22	4.30	3.62	3.71
		0.5	92.74	20.86	17.32	17.51	16.03	16.28	36.82	6.93	4.97	5.06	4.58	4.69
	0.9	0.1	146.28	71.94	69.17	69.33	66.74	67.02	97.33	36.83	32.64	32.87	31.10	31.40
		0.3	148.13	74.82	72.14	72.29	69.71	69.99	100.97	39.79	35.59	35.83	33.96	34.26
		0.5	151.36	80.27	77.77	77.91	75.37	75.64	107.00	45.27	41.10	41.35	39.31	39.62
500	0.5	0.1	64.62	3.12	1.75	1.81	1.53	1.58	5.96	1.05	0.70	0.70	0.65	0.68
		0.3	67.96	3.27	1.86	1.92	1.64	1.70	6.72	1.13	0.72	0.73	0.66	0.67
		0.5	74.38	3.79	2.11	2.18	1.91	1.97	8.28	1.29	0.77	0.79	0.69	0.70
	0.6	0.1	110.36	6.40	4.13	4.25	3.77	3.92	16.85	1.80	1.10	1.11	0.91	0.93
		0.3	115.21	6.96	4.52	4.65	4.14	4.29	18.88	1.96	1.19	1.20	0.96	1.00
		0.5	124.36	8.12	5.39	5.52	4.93	5.11	22.86	2.23	1.38	1.40	1.11	1.15
	0.75	0.1	213.17	27.66	21.91	22.22	20.00	20.39	68.84	7.25	4.92	5.05	4.51	4.66
		0.3	219.51	29.94	24.00	24.33	21.92	22.33	74.89	8.19	5.63	5.77	5.27	5.44
		0.5	231.04	34.69	28.41	28.76	25.99	26.44	85.93	10.17	7.16	7.31	6.94	7.15
	0.9	0.1	365.61	146.74	138.28	138.73	132.83	133.56	237.52	68.67	59.76	60.22	58.57	59.20
		0.3	370.26	153.77	145.38	145.83	139.89	140.63	246.74	75.11	65.95	66.43	64.70	65.35
		0.5	378.37	167.25	159.04	159.48	153.52	154.25	262.01	87.27	77.76	78.26	76.47	77.15

**Table 11:** Phase-I dataset for the example.

Sample number, t	Sample means		Sample variances and covariances			${\hat{\gamma }}_{t}$
	${\overset{―}{X}}_{1t}$	${\overset{―}{X}}_{2t}$	$S_{1t}^{2}$	$S_{2t}^{2}$	$S_{12t}$	
1	9.896	2.087	1.423	1.396	0.124660	0.119630
2	9.930	1.900	1.758	1.121	−0.520450	0.111590
3	9.230	0.913	1.270	0.116	0.161240	0.121460
4	10.043	2.215	0.821	1.327	0.187860	0.090222
5	10.244	1.280	0.641	1.243	−0.287520	0.071698
6	9.625	2.645	2.022	0.375	0.261490	0.139250
7	9.578	2.727	0.767	0.824	−0.544030	0.055312
8	10.654	3.031	0.947	0.781	0.069420	0.088955
9	9.940	1.439	0.709	1.626	−0.272150	0.079670
10	9.124	2.716	0.858	0.055	−0.155290	0.035141
11	9.251	1.726	0.629	0.787	−0.290230	0.072364
12	9.528	2.587	0.981	0.070	−0.044020	0.066513
13	9.697	2.412	0.675	1.551	−0.455280	0.070077
14	9.372	.2.603	0.243	0.669	0.079130	0.052576
15	11.005	1.635	2.043	0.602	−0.614910	0.092041
16	9.555	1.780	0.524	2.595	−0.031450	0.075270
17	9.724	2.526	1.257	0.228	0.179020	0.110680
18	9.952	2.239	0.697	0.895	0.330970	0.081546
19	9.511	2.155	1.845	0.838	−0.440540	0.114880
20	10.386	1.852	0.570	0.477	−0.325140	0.050179

The 4 regions VSI RS MCV chart will be optimally designed for identifying process MCV shifts, by minimizing ATS(*τ*), based on *τ* = 1.25 and 0.75, for the upward and downward charts, respectively, when $\text{AT}{\text{S}}_{\text{0}}$= 200, ${\hat{\gamma }}_{\text{0}}$ = 0.089115, $d_{\text{0}}$ = 1 hour, *G* = 4 and $d_{\text{1}}$ = 0.1 hour are employed. Consequently, the optimal parameter and score combination $\left(d_{\text{2}},K,\left\{\pm S_{\text{1}},\pm S_{\text{2}},\pm S_{\text{3}},\pm S_{\text{4}}\right\}\right)$, computed using the MATLAB optimization programs are (1.2770, 1.0046,{0, 1, 2, 4}) and(1.3613, 0.7490,  {−1, −2,−2,−8}), for the upward and downward VSI RS MCV charts, respectively. Equation (18) is used to calculate the upper control limits of the upward VSI RS MCV chart as ${\text{UCL}}_{\text{0}}=\text{0}.\text{0685}$, ${\text{UCL}}_{\text{1}}=\text{0}.\text{1021}$, ${\text{UCL}}_{\text{2}}=\text{0}.\text{1391}$ and ${\text{UCL}}_{\text{3}}=\text{0}.\text{1792}$, while Equation (19) is used to compute the lower control limits of the downward VSI RS MCV chart, which gives ${\text{LCL}}_{\text{0}}$ = 0.0685, ${\text{LCL}}_{\text{1}}=\text{0}.\text{0304}$, ${\text{LCL}}_{\text{2}}=\text{0}.\text{0150}$ and ${\text{LCL}}_{\text{3}}=\text{0}.\text{0057}$.

The length of the sampling interval used in taking the next sample is determined using Equation (17). The short sampling interval is specified as $d_{\text{1}}$ = 0.1 hour and the corresponding long sampling intervals are computed from the MATLAB optimization programs for the upward and downward charts as $d_{\text{2}}$ = 1.2770 hours and 1.3613 hours, respectively. For the upward chart, the triggering score is $+S_{\text{4}}$ = + 4. As *G* = 4, then $+S_{\text{4}}/G$ = + 4/4 = +1, hence, if 0 ≤ $U_{t}$ <+1, the upward chart requires taking the next sample after the long sampling interval, i.e., $d_{\text{2}}$ = 1.2770 hours. Conversely, if +1 ≤ $U_{t}$ <+4, the upward chart requires taking the next sample after the short sampling interval, i.e., $d_{\text{1}}$ = 0.1 hour. On similar lines, for the downward chart, the triggering score is $-S_{\text{4}}$ = −8. Since *G* = 4, then $-S_{\text{4}}/G$ = −8/4 = −2. Therefore, if −2 <$L_{t}$ ≤ 0, the long sampling interval $d_{\text{2}}$ = 1.3613 hours is used to take the next sample. However, if −8 <$L_{t}$ ≤ −2, the short sampling interval $d_{\text{1}}$ = 0.1 hour is used to take the next sample for the downward chart. Also, $U_{\text{0}}$ = $L_{\text{0}}$ = 0 is adopted in constructing the 4 regions VSI RS MCV chart.

**Fig 3:**
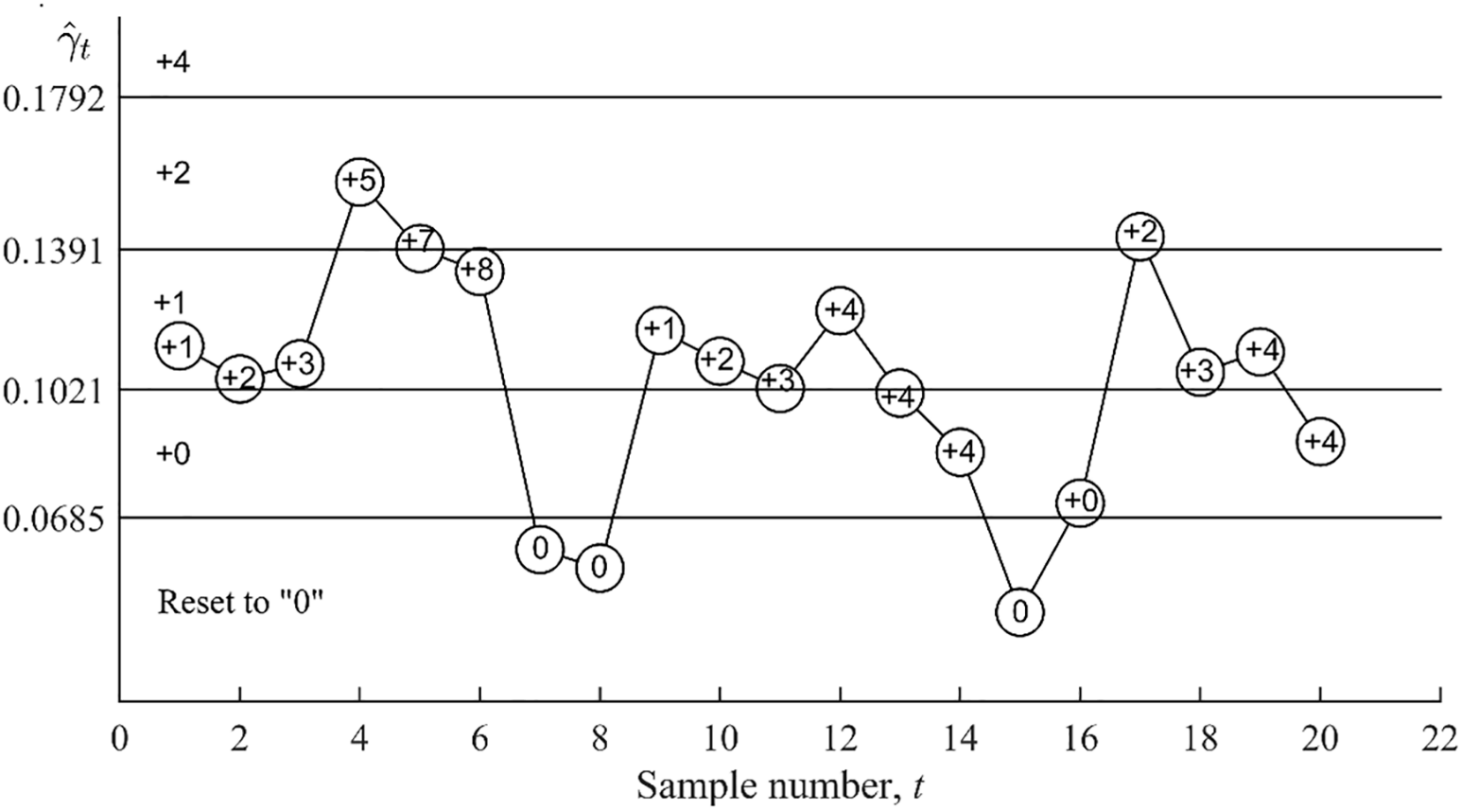
Implementation of the upward VSI RS MCV chart.

**Fig 4:**
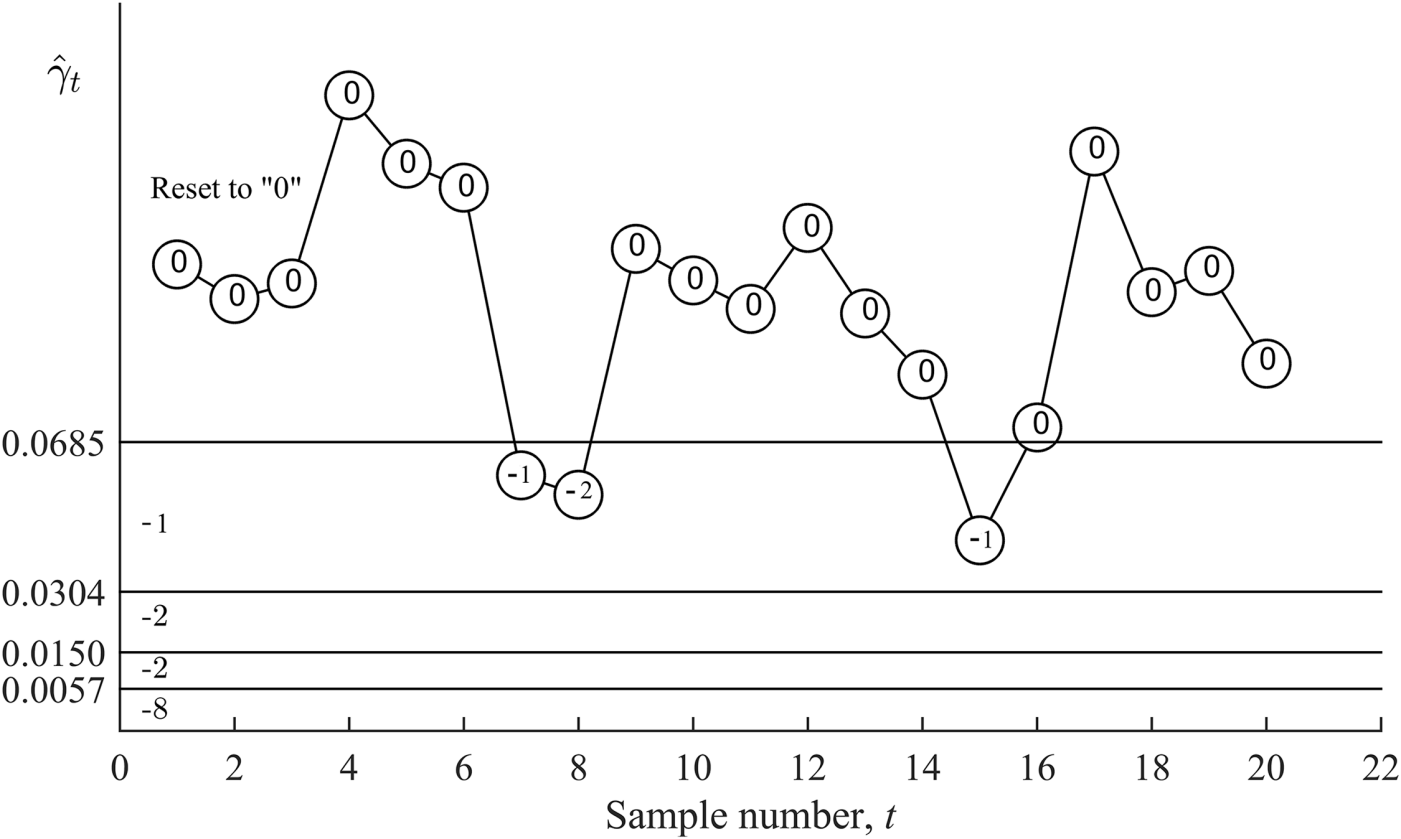
Implementation of the downward VSI RS MCV chart.

Table 12 gives the Phase-II dataset, as well as the values of the computed ${\hat{\gamma }}_{t}$, $S\left({\hat{\gamma }}_{t}\right)$, $U_{t}$, $L_{t}$, $d_{\text{1}}$/$d_{\text{2}}$ and elapsed time. There are 20 samples in the Phase-II dataset, each having a sample size *n* = 5. An explanation of the working of the upward VSI RS MCV chart is given henceforth. At sample 1 (*t* = 1), $\;{\hat{\gamma }}_{\text{1}}=\text{0}.\text{11371}\in {(\text{UCL}}_{\text{1}},{\text{UCL}}_{\text{2}})$ = $R_{+\text{2}}$ is obtained, thus, the score $S\left({\hat{\gamma }}_{\text{1}}\right)=+\text{1}$ is assigned and the cumulative score becomes $U_{\text{1}}=U_{\text{0}}+S\left({\hat{\gamma }}_{\text{1}}\right)$ = 0 + 1 = +1. Consequently, sample 2 (*t* = 2) is taken after the short sampling interval $d_{\text{1}}$ = 0.1 hour, as +1 ≤ $U_{\text{1}}$ <+4. At *t* = 2, $\;{\hat{\gamma }}_{\text{2}}=\text{0}.\text{10489}\in {(\text{UCL}}_{\text{1}},{\text{UCL}}_{\text{2}})$ = $R_{+\text{2}}$ is obtained, thus, the score $S\left({\hat{\gamma }}_{\text{2}}\right)=+\text{1}$ is assigned and the cumulative score becomes $U_{\text{2}}=U_{\text{1}}+S\left({\hat{\gamma }}_{\text{2}}\right)$ = + 1 + 1 = +2. Since +1 ≤ $U_{\text{2}}$ <+4, the short sampling interval $d_{\text{1}}$ = 0.1 hour is employed to *t*ake the third sample (*t* = 3). The process of taking samples and updating the cumulative score, $U_{t}$, by adding the most recent score continues until sample 4. Considering that +1 ≤ $U_{\text{3}}\;(=+\text{3})$ <+4 (see Table 12), sample 4 is taken by using the short sampling interval $d_{\text{1}}=\text{0}.\text{1}$ hour. As ${\hat{\gamma }}_{\text{4}}=\text{0}.\text{15679}\in$ $(\;{\text{UCL}}_{\text{2}},{\text{UCL}}_{\text{3}})$ = $R_{+\text{3}}$, thus the score $S\left({\hat{\gamma }}_{\text{4}}\right)=+\text{2}$ is assigned and the cumulative score $U_{\text{4}}=+\text{5}$. The upward VSI RS MCV char*t* will issue an out-of-control signal if the cumulative score reaches or exceeds the triggering score of +4. Thus, at sample 4, the upward VSI RS MCV chart issues the first out-of-control signal. The duration taken from *t*he start of process monitoring to giving this out-of-control signal is 0.3 hours (see Table 12). Fig 3 and 4 plot the upward and downward VSI RS MCV charts and show the cumulative scores $U_{t}$ and $L_{t}$, respectively, for *t* = 1, 2, …, 20, in Table 12.

**Table 12:** Phase-II dataset for the example.

Sample number	Sample means		Sample variances and covariances				Upward VSI RS MCV				Downward VSI RS MCV			
t	${\overset{―}{X}}_{1t}$	${\overset{―}{X}}_{2t}$	$S_{1t}^{2}$	$S_{2t}^{2}$	$S_{12t}$	${\hat{\gamma }}_{t}$	$S({\hat{\gamma }}_{t})$	Cumulative score, $U_{t}$	Sampling interval $d_{1}$ or$d_{2}$	Total time elapsed	$S({\hat{\gamma }}_{t})$	Cumulative Score, $L_{t}$	Sampling interval $d_{1}$ or$d_{2}$	Total time elapsed
1	7.781	1.592	1.164	0.734	0.35645	0.11371	+1	+1	0.1		0	Reset to “0”	1.3613	
2	7.385	1.804	1.006	1.337	0.96049	0.10489	+1	+2	0.1	0.1000	0	Reset to “0”	1.3613	1.3613
3	7.988	2.26	0.762	0.359	0.17373	0.10887	+1	+3	0.1	0.2000	0	Reset to “0”	1.3613	2.7226
4	8.189	2.1	1.885	0.47	0.13026	0.15679	+2	+5	0.1	0.3000	0	Reset to “0”	1.3613	4.0839
5	7.436	2.061	1.404	0.519	0.08280	0.13929	+2	+7	0.1	0.4000	0	Reset to “0”	1.3613	5.4452
6	6.746	2.289	0.846	0.811	0.43835	0.13324	+1	+8	0.1	0.5000	0	Reset to “0”	1.3613	6.8065
7	7.356	1.917	0.197	2.587	0.01597	0.05999	0	Reset to “0”	1.2770	0.6000	−1	−1	1.3613	8.1678
8	8.492	1.845	1.46	1.746	1.42051	0.05509	0	Reset to “0”	1.2770	1.8770	−1	−2	0.1	9.5291
9	7.272	1.58	1.353	0.345	0.27988	0.11771	+1	+1	0.1	3.1540	0	Reset to “0”	1.3613	9.6291
10	7.585	1.568	1.098	0.788	0.41252	0.10961	+1	+2	0.1	3.2540	0	Reset to “0”	1.3613	10.9904
11	7.734	1.709	0.952	0.228	0.11462	0.10244	+1	+3	0.1	3.3540	0	Reset to “0”	1.3613	12.3517
12	8.16	1.498	1.598	1.178	1.00757	0.12295	+1	+4	0.1	3.4540	0	Reset to “0”	1.3613	13.7130
13	7.102	2.661	1.508	0.945	0.73607	0.10126	+0	+4	0.1	3.5540	0	Reset to “0”	1.3613	15.0743
14	8.392	1.883	0.536	0.706	0.23234	0.08564	+0	+4	0.1	3.6540	0	Reset to “0”	1.3613	16.4356
15	7.592	2.531	0.256	0.563	0.24827	0.04349	0	Reset to “0”	1.2770	3.7540	−1	−1	1.3613	17.7969
16	8.141	2.093	0.394	0.603	0.25584	0.07220	+0	+0	1.2770	5.0310	0	Reset to “0”	1.3613	19.1582
17	7.883	2.49	1.321	1.179	0.65037	0.14243	+2	+2	0.1	6.3080	0	Reset to “0”	1.3613	20.5195
18	7.886	2.877	0.883	1.431	0.22524	0.10668	+1	+3	0.1	6.4080	0	Reset to “0”	1.3613	21.8808
19	7.83	1.008	0.878	0.558	0.14223	0.11209	+1	+4	0.1	6.5080	0	Reset to “0”	1.3613	23.2421
20	8.196	1.482	0.791	0.22	0.13724	0.08846	+0	+4	0.1	6.6080	0	Reset to “0”	1.3613	24.6034

For the downward VSI RS MCV chart, as ${\hat{\gamma }}_{\text{1}}=$ $\;\text{0}.\text{11371}\in {(\text{LCL}}_{\text{0}},\infty )$ = $R_{\text{0}}$, the lower cumulative score is reset to zero, i.e., $L_{\text{1}}$ = 0. Since −2 <$L_{\text{1}}$ ≤ 0, sample 2 is taken after the long sampling interval $d_{\text{2}}=\text{1}.\text{3613}$ hours. The process of taking samples and updating the cumulative score, $L_{t}$, by adding the most recent score continues until sample 7. At sample 7, ${\hat{\gamma }}_{\text{7}}=$ $\;\text{0}.\text{05999}\in {({\text{LCL}}_{\text{1}},\text{LCL}}_{\text{0}})$ = $R_{-\text{1}}$ is obtained, thus, the score $S\left({\hat{\gamma }}_{\text{7}}\right)=-\text{1}$ is added to the cumulative score $L_{\text{6}}$ and consequently, resulting in $L_{\text{7}}=-\text{1}$. Since −2 <$L_{\text{7}}$ ≤ 0, thus, the long sampling interval $d_{\text{2}}=\text{1}.\text{3613}$ hours is used to take sample 8. The above-mentioned process continues until sample 20 (*t* = 20). Since none of the 20 samples produce $L_{t}\leq -\text{8}$, the downward VSI RS MCV chart does not give any out-of-control signal.

## 7. Conclusions

This paper proposed the VSI RS MCV chart, which enhances the MCV chart’s performance by integrating the VSI and RS methodologies. The VSI RS MCV chart is compared with the existing MCV and RS MCV charts. The findings indicate that the proposed VSI RS MCV chart surpasses the two existing charts under comparison for all sizes of process MCV shifts, based on the ATS, SDTS and EATS criteria. The efficacy of the proposed VSI RS MCV chart improves with a shorter sampling interval $d_{\text{1}}$, a higher switching rate *G* or a bigger sample size *n*, as indicated by the lower ATS(*τ*), SDTS(*τ*) and $\text{EATS}\left(\tau_{\text{min}},\tau_{\text{max}}\right)$ values. Another important conclusion is that the ATS(*τ*), SDTS(*τ*) and $\text{EATS}\left(\tau_{\text{min}},\tau_{\text{max}}\right)$ values decrease when $\gamma_{\text{1}}$ deviates further from $\gamma_{\text{0}}$. This suggests that larger shift sizes in the process MCV can be detected quicker.

Future research may include the concurrent application of the variable sample size and sampling interval features to further improve the efficacy of the RS MCV control chart. Another research direction is the development of adaptive MCV charts using machine learning, which is facilitated by the advancement of artificial intelligence. Moreover, as the majority of existing MCV control charts continue to rely on the premise of multivariate normal distribution, future study may concentrate on developing robust MCV control charts based on multivariate non-normal distributions, such as the multivariate gamma or *t* distribution.

## References

[1] Kang CW, Lee MS, Seong YJ, Hawkins DM. A control chart for the coefficient of variation. J Qual Technol. 2007;39(2):151–8. doi: 10.1080/00224065.2007.11917682

[2] Ayesha S, Arshad A, Albalawi O, Alharthi AM, Hanif M, Yasmeen U, et al. New adaptive EWMA CV control chart with application to the sintering process. Sci Rep. 2024;14(1):11565. doi: 10.1038/s41598-024-62316-4 38773191 PMC11109341

[3] Yeong WC, Khoo MBC, Teoh WL, Castagliola P. A control chart for the multivariate coefficient of variation. Qual Reliab Eng. 2015;32(3):1213–25. doi: 10.1002/qre.1828

[4] Abbasi SA, Adegoke NA. Multivariate coefficient of variation control charts in phase I of SPC. Int J Adv Manuf Technol. 2018;99(5–8):1903–16. doi: 10.1007/s00170-018-2535-3

[5] Khaw KW, Khoo MBC, Castagliola P, Rahim MA. New adaptive control charts for monitoring the multivariate coefficient of variation. Comput Ind Eng. 2018;126:595–610. doi: 10.1016/j.cie.2018.10.16

[6] Chew X, Khoo MBC, Khaw KW, Yeong WC, Chong ZL. A proposed variable parameter control chart for monitoring the multivariate coefficient of variation. Qual Reliab Eng. 2019;35(7):2442–61. doi: 10.1002/qre.2536

[7] Yeong WC, Lim SL, Chong ZL, Khoo MBC, Saha S. A side-sensitive synthetic chart for the multivariate coefficient of variation. PLoS One. 2022;17(7):e0270151. doi: 10.1371/journal.pone.0270151 35788210 PMC9255772

[8] Saha S, Khoo MBC, Babatunde OT, Teh SY, Teoh WL. Run sum hotelling’s T2${T}^2$ chart for autocorrelated processes. Qual Reliab Eng. 2025;41(5):2147–63. doi: 10.1002/qre.3775

[9] Hu X, Ma Y, Zhang J, Zhang J, Yeganeh A, Shongwe SC. The efficiency of CUSUM schemes for monitoring the multivariate coefficient of variation in short runs process. J Appl Stat. 2024;52(4):966–92. doi: 10.1080/02664763.2024.2405111 40040676 PMC11873948

[10] Champ CW, Rigdon SE. An analysis of the run sum control chart. J Qual Technol. 1997;29(4):407–17. doi: 10.1080/00224065.1997.11979792

[11] Rakitzis AC, Antzoulakos DL. Run sum control charts for the monitoring of process variability. Qual Technol Quant Manag. 2016;13(1):58–77. doi: 10.1080/16843703.2016.1139842

[12] Teoh WL, Khoo MBC, Castagliola P, Yeong WC, Teh SY. Run-sum control charts for monitoring the coefficient of variation. Eur J Oper Res. 2017;257(1):144–58. doi: 10.1016/j.ejor.2016.08.067

[13] Lim AJX, Khoo MBC, Teoh WL, Haq A. Run sum chart for monitoring multivariate coefficient of variation. Comput Ind Eng. 2017;109:84–95. doi: 10.1016/j.cie.2017.04.023

[14] Le Goh K, Teoh WL, Chong ZL, Ong KL, El-Ghandour L. A study on the performances of the run sum $\bar{X}$ chart under the gamma process. ITM Web Conf. 2024;67:01002. doi: 10.1051/itmconf/20246701002

[15] Teoh WL, Teoh JW, Goh KL, Song Z, Saha S. Enhanced designs for the multi‐region run sum $\bar{X}$ control chart based on the median run length metric. Qual Reliab Eng. 2025;41(4):1362–85. doi: 10.1002/qre.3721

[16] Reynolds MR, Amin RW, Arnold JC, Nachlas JA. $\bar{X}$ charts with variable sampling intervals. Technometrics. 1988;30(2):181–92. doi: 10.1080/00401706.1988.10488366

[17] Saccucci MS, Amin RW, Lucas JM. Exponentially weighted moving average control schemes with variable sampling intervals. Commun Stat Simul Comput. 1992;21(3):627–57. doi: 10.1080/03610919408813291

[18] Baxley RV Jr. An application of variable sampling interval control charts. J Qual Technol. 1995;27(4):275–82. doi: 10.1080/00224065.1995.11979607

[19] Costa AFB. $\bar{X}$ chart with variable sample size and sampling intervals. J Qual Technol. 1997;29(2):197–204. doi: 10.1080/00224065.1997.11979750

[20] Hu X, Castagliola P, Sun J, Khoo MBC. Effect of measurement errors on the VSI $\bar{X}$ chart. EJIE. 2016;10(2):224. doi: 10.1504/ejie.2016.075853

[21] Yeong WC, Khoo MBC, Tham LK, Teoh WL, Rahim MA. Monitoring the coefficient of variation using a variable sampling interval EWMA chart. J Qual Technol. 2017;49(4):380–401. doi: 10.1080/00224065.2017.11918004

[22] Ng PS, Khoo MBC, Saha S, Lee MH. A variable sampling interval EWMA t chart with auxiliary information – a robustness study in the presence of estimation error. Alex Eng J. 2022;61(8):6043–59. doi: 10.1016/j.aej.2021.11.033

[23] Chew XY, Khoo MBC, Teh SY, Castagliola P. The variable sampling interval run sum $\bar{X}$ control chart. Comput Ind Eng. 2015;90:25–38. doi: 10.1016/j.cie.2015.08.015

[24] Yeong WC, Lim SL, Khoo MBC, Ng PS, Chong ZL. A variable sampling interval run sum chart for the coefficient of variation. J Stat Comput Simul. 2022;92(15):3150–66. doi: 10.1080/00949655.2022.2061486

[25] Yeong WC, Tan YY, Lim SL, Khaw KW, Khoo MBC. Variable sample size and sampling interval (VSSI) and variable parameters (VP) run sum charts for the coefficient of variation. Qual Technol Quant Manag. 2023;21(2):177–99. doi: 10.1080/16843703.2023.2177812

[26] Antzoulakos DL, Fountoukidis KG, Rakitzis AC. The variable sample size and sampling interval run sum Max chart. Qual Technol Quant Manag. 2024;22(2):321–44. doi: 10.1080/16843703.2024.2315837

[27] Voinov VG, Nikulin MS. Unbiased estimators and their applications: multivariate case. Dordrecht: Kluwer; 1996. doi: 10.1007/978-94-009-0289-7

[28] Wijsman RA. Random orthogonal transformations and their use in some classical distribution problems in multivariate analysis. Ann Math Statist. 1957;28(2):415–23. doi: 10.1214/aoms/1177729350

[29] Saha S, Khoo MBC, Ng PS, Chong ZL. Variable sampling interval run sum median charts with known and estimated process parameters. Comput Ind Eng. 2019;127:571–87. doi: 10.1016/j.cie.2018.10.049

[30] Saha S, Khoo MBC, Lee MH, Castagliola P. A side-sensitive modified group runs double sampling (SSMGRDS) control chart for detecting mean shifts. Commun Stat - Simul Comput. 2017;47(5):1353–69. doi: 10.1080/03610918.2017.1311918

[31] Prabhu SS, Montgomery DC, Runger GC. A combined adaptive sample size and sampling interval control scheme. J Qual Technol. 1994;26(2):164–76. doi: 10.1080/00224065.1994.11979232

